# Comparing Respiratory Support Modalities in Pediatric Asthma Exacerbation–A Systematic Review and Meta-Analysis

**DOI:** 10.3390/pediatric18040104

**Published:** 2026-08-04

**Authors:** Maha A. Odeh, Garam Kiswani, Alex Gileles-Hillel

**Affiliations:** 1 Pediatric Department, Hadassah Medical Center, Jerusalem 24035, Israel; mahaodeh1997@gmail.com; 2 Department of Family Medicine, The Hebrew University, Jerusalem 12272, Israel; 3 Pediatric Pulmonology and Sleep Unit, Hadassah Medical Center, Jerusalem 12000, Israel; 4 Faculty of Medicine, The Hebrew University of Jerusalem, Jerusalem 12272, Israel

**Keywords:** high-flow, non-invasive ventilation, invasive ventilation, asthma exacerbations, racial disparities

## Abstract

**Objectives:** To compare clinical outcomes and racial disparities of children hospitalized for acute asthma exacerbation who required any respiratory support: invasive mechanical ventilation (IMV), non-invasive modalities (non-invasive ventilation (NIV) and high-flow nasal cannula (HFNC)). **Methods:** We searched PubMed, Embase, Cochrane, and Scopus for randomized controlled trials (RCTs) and observational studies published between 2010 and 2025 that involved pediatric asthma patients (0–18 years) who received HFNC, NIV, or IMV. Network meta-analyses (NMA) were conducted separately for RCTs (change in asthma score) and observational studies (PICU length of stay). Subgroup analyses compared respiratory support modalities and failure rates. Racial and ethnic disparities were analyzed narratively. **Results:** In five RCTs (*n* = 233), compared to oxygen, HFNC showed no significant benefit (MD = 0.24; *p* = 0.58), whereas NIV showed the greatest improvement in asthma scores (mean difference [MD] = 1.24; *p* = 0.07), reaching significance in sensitivity analysis (MD = 2.5; *p* < 0.001). Observational NMA found no differences in PICU stay between respiratory support modalities, but HFNC was associated with 2-fold increase in PICU stay in a subgroup analysis compared to standard oxygen (*p* = 0.04) and with a higher failure rate compared to NIV (12.6% vs. 2.6%; OR = 5.3, *p* < 0.001). Black children had higher odds of intubation. **Conclusions:** Available evidence suggests that NIV may confer greater short-term clinical benefit in children with severe acute asthma requiring respiratory support, although findings should be interpreted cautiously given the limited and heterogeneous data. Further high-quality studies with standardized outcomes are needed to inform respiratory support selection.

## 1. Introduction

Acute asthma exacerbations are a leading cause of pediatric emergency department visits and hospital admissions [1]. In 2022, pediatric patients accounted for over a quarter of all asthma-related emergency department visits and hospitalizations in the United States, with associated annual costs exceeding 480 $ million [2,3].

Most exacerbations respond to standard therapy, including inhaled bronchodilators, systemic corticosteroids, and low-flow oxygen. A small subset of children with more severe asthma may require advanced respiratory support, such as high-flow nasal cannula (HFNC), non-invasive ventilation (NIV), or invasive mechanical ventilation (IMV) [4]. The use of non-invasive modalities, particularly HFNC and NIV, has been on the rise, owing in part to the known adverse events associated with IMV [5]. A meta-analysis in adults with asthma concluded that compared to standard therapy, NIV may accelerate clinical improvement and reduce intubation rates [6]. The evidence supporting the efficacy of HFNC and NIV in children with asthma remains unclear, with most studies characterized by small sample sizes and methodological limitations [7].

Beyond these clinical uncertainties, racial and ethnic disparities significantly affect the outcomes of asthma exacerbations in children, reflecting broader socioeconomic inequalities. For example, compared to other racial and ethnic groups, Black children have the highest rates of asthma-related hospitalization and intubation rates [8,9].

To date, no systematic review has compared the effectiveness and safety of respiratory support modalities in the pediatric asthma population. In this review and network meta-analyses, we examined the use, effectiveness, and safety of HFNC, NIV, and IMV. Primary outcomes included hospitalization duration and pediatric intensive care unit (PICU) length of stay, changes in vital signs and asthma severity scores, ventilation failure, and mortality. Secondary outcomes included racial and ethnic disparities in modality use and outcomes.

## 2. Materials and Methods

This systematic review was registered in PROSPERO Centre for Reviews and Dissemination, University of York, York, UK, and the protocol is available from https://www.crd.york.ac.uk/PROSPERO/view/CRD420251032100 (accessed on 14 April 2025). Amendments clarifying the definition of treatment effectiveness were made before data analysis and are documented in the PROSPERO record. The review adhered to the Preferred Reporting Items for Systematic Reviews and Meta-Analyses (PRISMA) guidelines.

### 2.1. Search Strategy and Study Criteria

**Figure 1:**
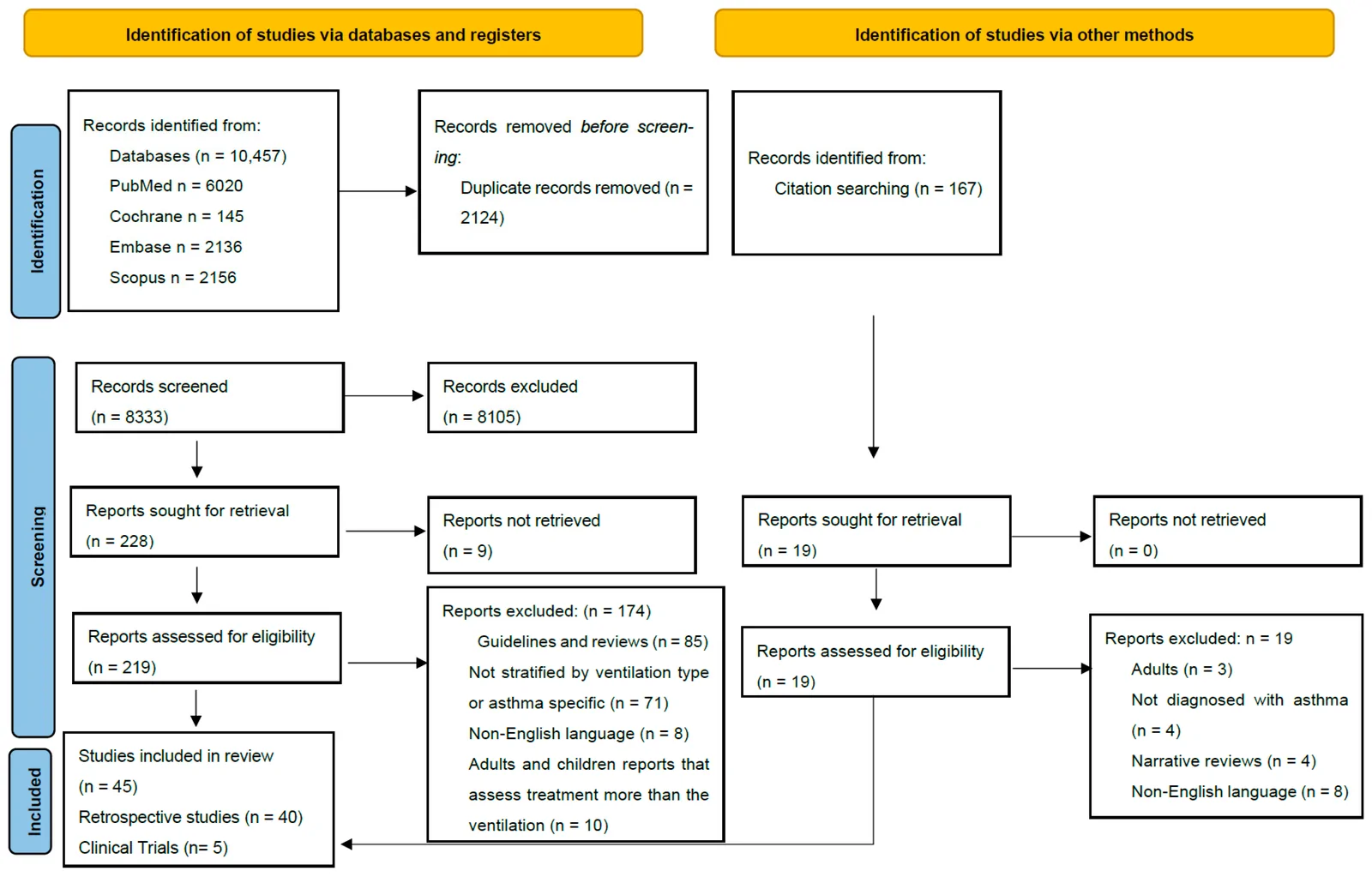
PRISMA flow chart. PRISMA, Preferred Reporting Items for Systematic Reviews and Meta-Analyses.

A systematic search was conducted in March 2025 across PubMed, Embase, Cochrane, and Scopus for studies examining respiratory support modalities in acute pediatric asthma exacerbations. Eligible studies included RCTs, cohort studies (retrospective and prospective), and cross-sectional studies (Figure 1). The target population was limited to pediatric patients diagnosed with asthma exacerbation, predominantly aged from 0 to 18 (one study included patients up to 21 years).

The full search string and filters are available in the Supplementary Materials. The search was restricted to articles published between 1 January 2010, and 1 February 2025. Table 1 and Table 2 specify the main characteristics of the studies included in the review. Additional demographic data are presented in the Supplementary Material.

### 2.2. Study Selection

Two reviewers (MAO and GK) independently screened titles and abstracts; the third reviewer (AGH) resolved any potential disagreements. Full texts of potentially eligible studies were reviewed. Additional relevant studies were identified through citation tracking.

### 2.3. Bias and Certainty Assessment

The certainty of evidence was independently assessed by two reviewers (MAO, GK) using the GRADE-pro GDT software Evidence Prime Inc., Hamilton, ON, Canada (accessed on 16 June 2025). GRADE summaries are provided in the Supplementary Materials [52]. Risk of bias in RCT was assessed using the Cochrane Risk of Bias (ROB 2) too (Version 2. Cochrane, London, UK), summarized in the Supplementary Data [53].

### 2.4. Statistical Analysis

The effectiveness of respiratory support modalities in the included RCTs was assessed using various asthma severity scores. Two studies used the Pulmonary Index Score (PIS), while three others used the Clinical Asthma Score (CAS), Pulmonary Score (PS), and Pediatric Respiratory Assessment Measure (PRAM) [54,55,56]. Although the scores range varied (0–9, 0–10, and 0–12), all three scores assessed similar clinical parameters (e.g., respiratory rate, wheezing, inspiratory to expiratory rate, and accessory muscle use), and were therefore conceptually comparable. However, due to imperfect data reporting across studies, a standardized mean difference (SMD) could not be calculated. This decision was made in consultation with a senior statistician to avoid introducing additional bias. Where data were missing or incompletely reported, numerical values were obtained directly from trial authors or estimated following Cochrane guidelines [10,57]. A random-effects network meta-analysis (NMA) model using restricted maximum likelihood (REML) estimation was implemented via the mvmeta package in StataBE 18 (Version 18; StataCorp LLC, College Station, TX, USA). Treatment effects were reported as MDs with 95% confidence intervals (CI) and *p* values. Ranking probabilities were calculated using the network rank command. Heterogeneity was assessed via the between-study standard deviation (τ) [58,59,60,61].

The primary results were derived from the consistency model, assuming agreement between direct (head-to-head trial) and indirect evidence (inferred by comparing each modality to a common comparator: standard oxygen). Network inconsistency (defined as disagreement between direct and indirect evidence) was formally evaluated. Where detected, sensitivity analyses were performed by sequentially excluding one study at time to identify the source, after which the model was re-evaluated to obtain a consistent network with the greatest accuracy.

A network meta-regression was conducted to examine whether treatment effects varied by follow-up time or by asthma score. However, the small number of RCTs prevented meaningful estimation of covariate effects [60]. Consequently, the primary random-effects consistency model without covariate adjustment was retained as the main analysis.

For observational studies reporting non-parametric data (e.g., PICU length of stay), medians were log-transformed, and standard deviations were estimated based on interquartile ranges or reported minimum-maximum ranges [57]. All outcomes were extracted from published data. Similar random-effects framework was applied [58,59].

No adjusted network meta-regression was feasible, because of the heterogenicity of reporting (asthma specific scores to physiologic or treatment proxies). This lack of standardization prevented formal transitivity or meta-regression adjustment, therefore residual confounding by indication possible.

Initially, we attempted to examine the total hospitalization length of stay (LOS), but due to the sparsity and inconsistency of available data, which were rarely stratified by ventilation modality, our analysis focused on PICU LOS. Pairwise subgroup meta-analyses compared HFNC vs. standard oxygen, NIV vs. HFNC, and intubated vs. non-intubated patients. Log-mean differences and back-transformed geometric mean ratios (GMRs), together with 95% CIs were reported. I_2_ statistics were used to quantify heterogeneity [62,63].

Publication bias was evaluated using funnel plots and Egger’s regression test.

Due to the limited ventilation-stratified data, racial and ethnic disparities are reported via narrative synthesis.

Temporal trends in the use of different respiratory support modalities were also examined and presented in the Supplementary Data (when numerical data were not provided, were extracted from published plots, using plot digitizer software Version 5.2; Ankit Rohatgi, Austin, TX, USA) [4,15,41,44,47,64,65].

Failure rates between HFNC and BiPAP were compared using Fisher’s exact test. Mortality differences across IMV, NIV, and HFNC were evaluated using a chi-squared test. A *p*-value < 0.05 was considered statistically significant.

All statistical analyses were performed in Stata/BE version 18 (StataCorp, LLC, College Station, TX, USA).

Terminology note: We recorded racial and ethnic classifications as originally reported in each study. For consistency, individuals described as African American, Black, or Non-Hispanic Black in the original studies are referred to as “Black” throughout the manuscript.

## 3. Results

### 3.1. Temporal Trends in Ventilation Use

Between 2000 and 2021, fourteen studies reported ventilation trends across three modalities: IMV, NIV, and HFNC [4,5,15,24,35,36,40,41,43,44,46,47,50,51]. Five studies included general hospitalized patients, and nine focused on PICU patients. The median of IMV use among hospitalized patients was 0.59% (IQR: 0.37–1.22%), showing a decline from 0.8% in 2000 to 0.47% in 2020. On the other hand, IMV use in PICU patients increased from 3.64% in 2000 to 8% in 2021, with a median of 9.75% (IQR: 4.5–14.75%).

Both settings showed increasing NIV use. Among hospitalized patients, median NIV use was 2.43% (IQR: 1.18–3.7%), rising from 0.14% in 2000 to 3.44% in 2020. In PICUs, NIV increased from 10.9% in 2000 to 16.5% in 2021 (median 5.5%, IQR: 1.22–12.37%). Three studies reported CPAP and BiPAP separately but were combined as NIV for this analysis [4,41,47].

**Figure 2:**
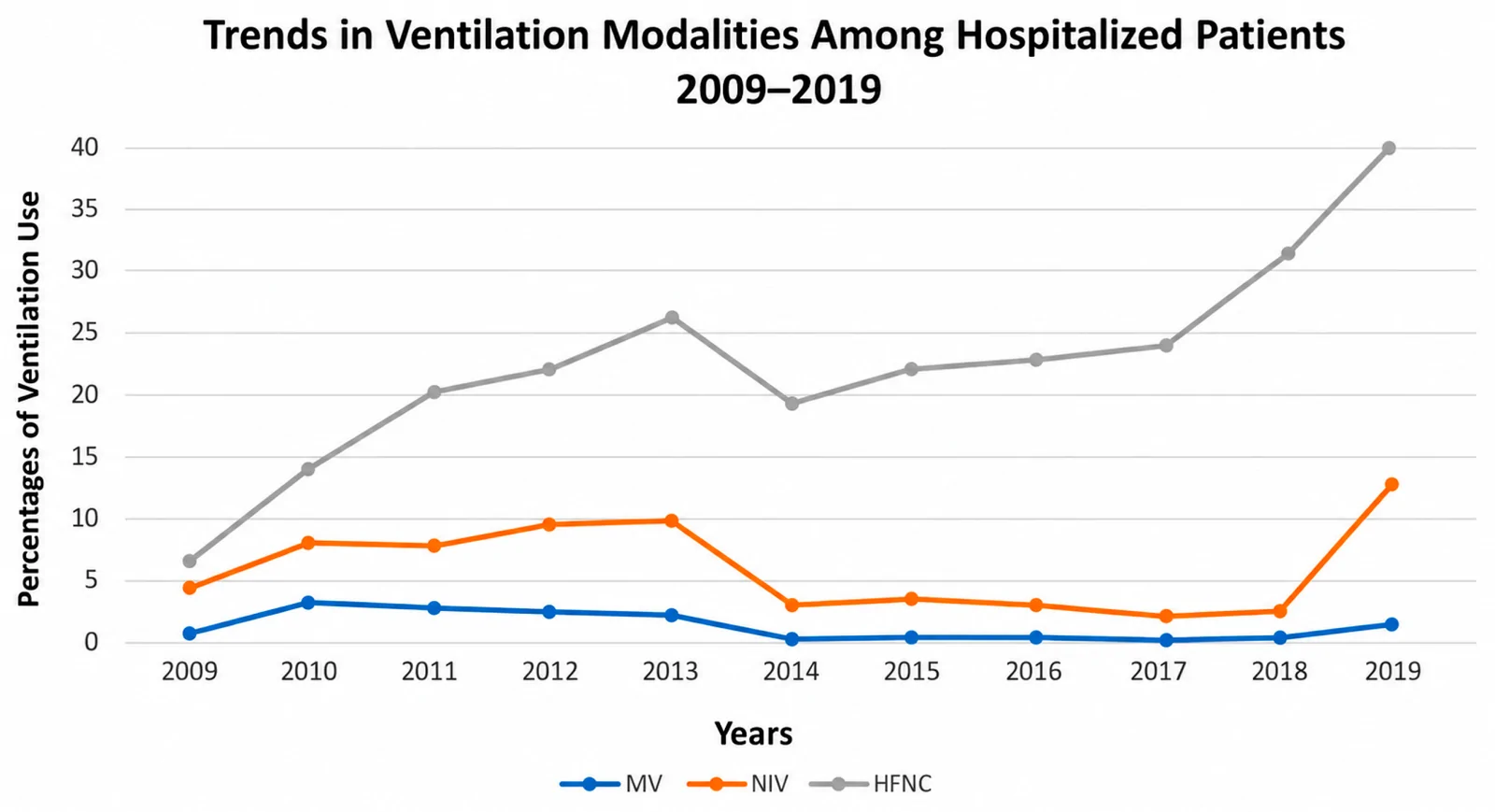
Trends in mechanical ventilation (IMV) *, non-invasive ventilation (NIV), and high-flow nasal cannula (HFNC) ** use among hospitalized pediatric patients from 2009 to 2019. Percentages represent the proportion of hospitalized patients receiving each modality. * IMV was never zero percent. ** HFNC data were reported in only one article.

**Figure 3:**
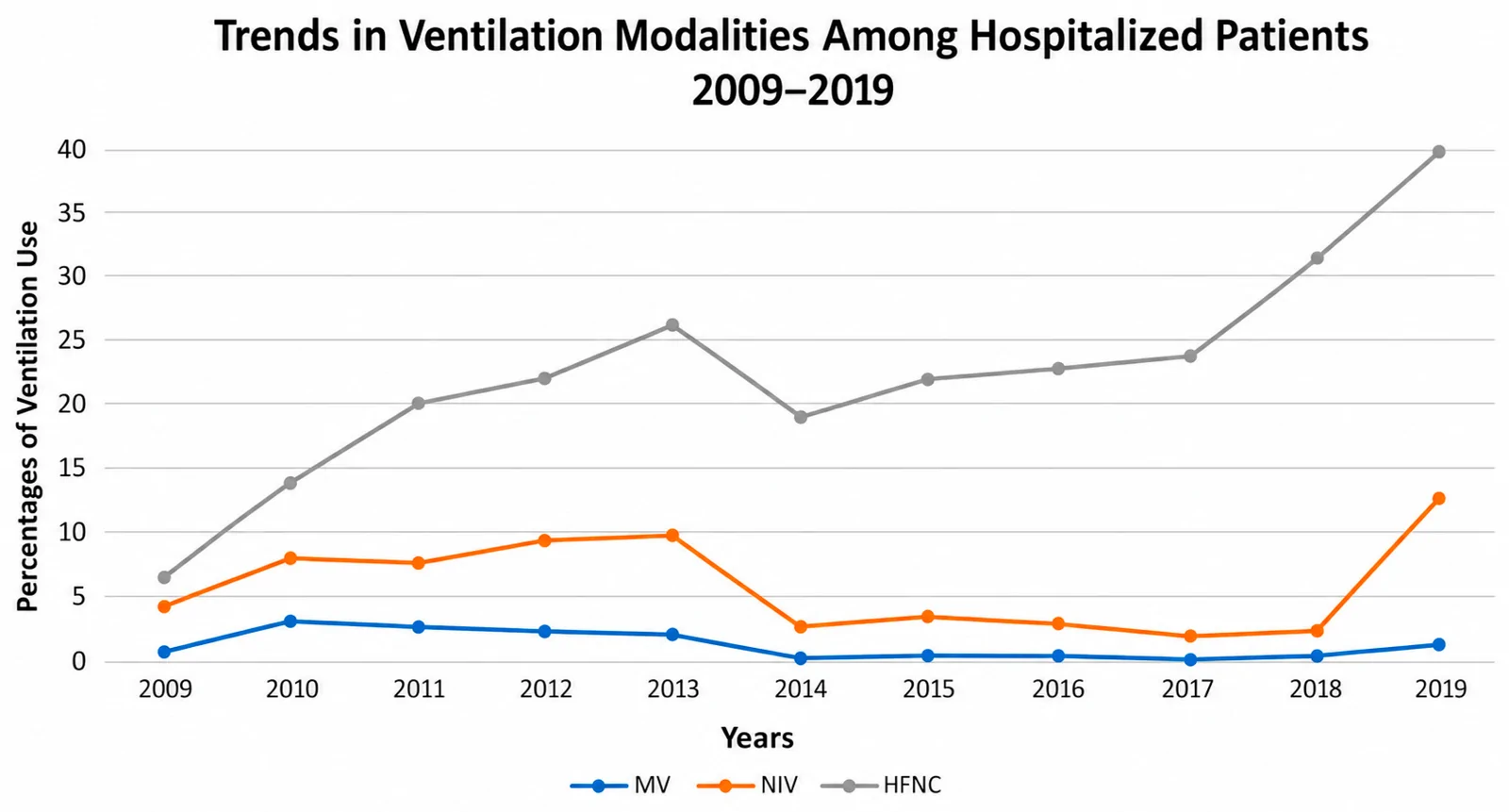
Trends in mechanical ventilation (IMV), non-invasive ventilation (NIV), and high-flow nasal cannula (HFNC) use among PICU patients from 2009 to 2019. Percentages represent the proportion of pediatric patients admitted to the PICU who received each ventilation modality.

HFNC use also rose substantially. Among hospitalized patients, HFNC use increased from 2.15% in 2009 to 34.29% in 2019 (median 16.33%, IQR: 12.43–20.70%), although this trend was reported in a single study [5]. In PICUs, HFNC use increased from 0% in 2004 to 62.94% in 2021 (median 13%, IQR: 0–27.07%). Figure 2 and Figure 3 illustrate these temporal patterns.

### 3.2. Network Meta-Analysis on Randomized Controlled Trials

All five eligible RCTs (n = 233 pediatric patients) were included in the network meta-analysis [10,11,12,13,14]. Across trials, most participants were early school-aged children, with reported means and medians ranging between 3 and 7 years. Asthma definitions were broadly similar, with minor differences in eligibility criteria. Children presented with moderate to severe exacerbations, with no significant differences between groups in baseline severity scores.

The three modalities compared were: NIV (studies using bilevel positive airway pressure (BiPAP) exclusively), HFNC, and standard oxygen. None of the included RCTs involved patients receiving IMV. The primary outcome across studies was improvement in various validated asthma scores measured after two hours of treatment. One study assessed outcome at 45 min and was included to generate direct evidence between BiPAP and HFNC, thereby closing the network [14]. The network structure is shown in the Supplementary Material.

**Figure 4:**
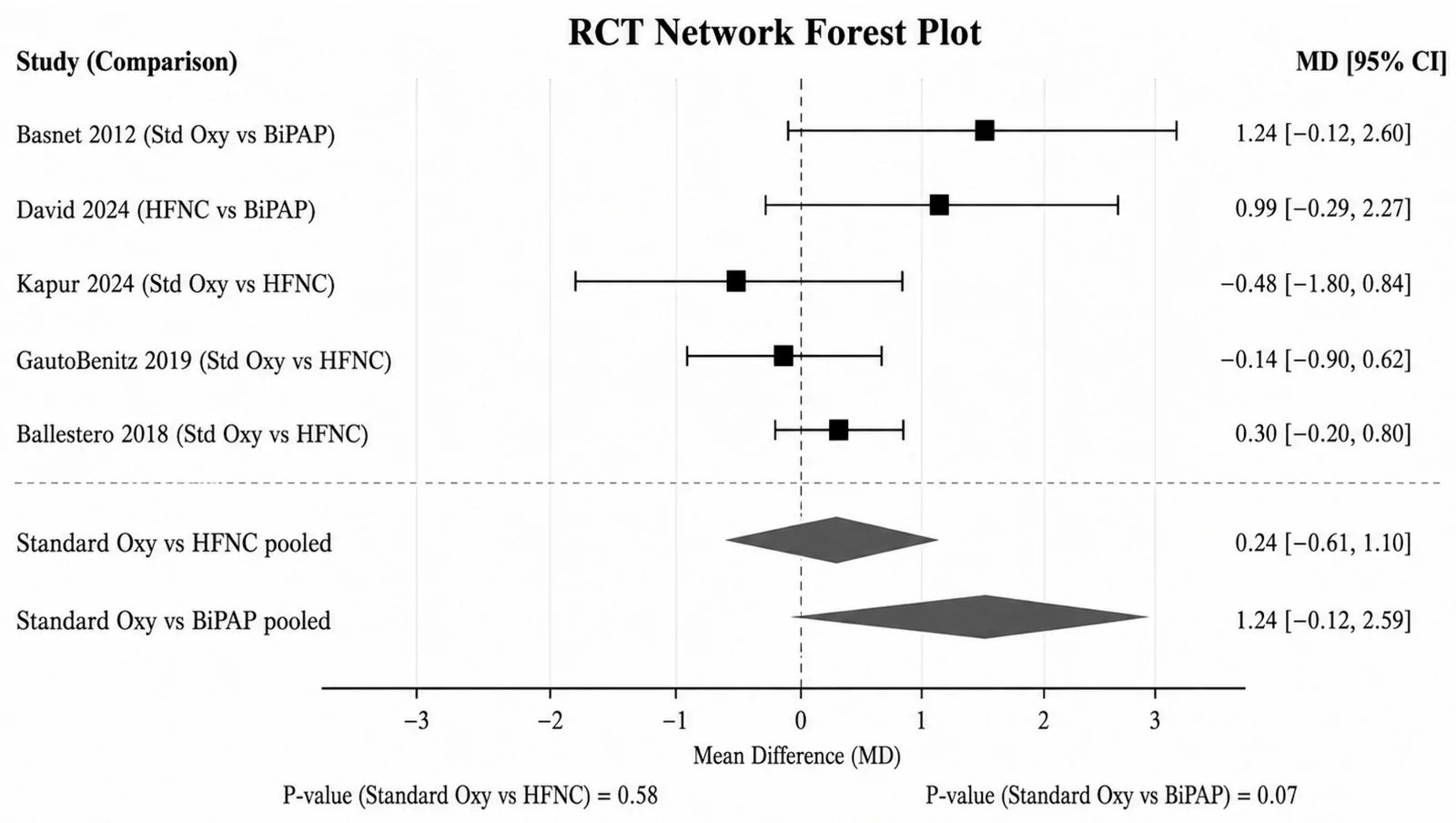
Forest plot of individual and polled RCT, evaluating BiPAP and HFNC compared to Standard Oxygen. Positive mean differences favor the second modality, and negative mean differences favor the first modality in each contrast. Squares indicate individual study effect estimates (mean differences), with horizontal lines representing 95% confidence intervals. Rhombuses indicate pooled network meta-analysis estimates, and their widths correspond to the 95% confidence intervals [10,11,12,13,14].

BiPAP demonstrated the greatest improvement in asthma scores compared to standard oxygen MD = 1.24 (95% CI −0.12 to 2.59; *p* = 0.07), which became statistically significant in the sensitivity analysis after excluding the study by David et al. [14] (see below). HFNC showed no difference compared to standard oxygen, MD = 0.24 (95% CI −0.61 to 1.1; *p* = 0.58). BiPAP was associated with a non-significant improvement in asthma score compared to HFNC with MD = 0.99 (95% CI −0.29 to 2.28; *p* = 0.13). These effects are illustrated in the Forest plot Figure 4.

BiPAP was the favored intervention according to ranking probabilities, followed by HFNC and standard oxygen, as detailed in the Supplementary Data Table. However, between-study heterogeneity was high (τ = 0.68), reflecting substantial variability in asthma scores and study populations. Although the funnel plot appeared asymmetric, Egger’s regression test did not reveal a statistically significant small study effect (*p* = 0.53). A significant inconsistency was detected in this network (*p* = 0.01). Excluding the Gauto Benítez et al., Ballestero et al., or Kapur et al. trials (separately) slightly reduced this inconsistency (*p* = 0.03, 0.01 and 0.01, respectively) [11,12,13], though it remained statistically significant. However, excluding either Basnet et al. or David et al. studies separately resolved the inconsistency entirely (*p* = 0.69 in both) [10,14]. This suggests that each of the two studies independently contributed to the observed inconsistency.

A sensitivity analysis followed. After excluding Basnet et al. study [10], BiPAP remained the top-ranked modality: for the BiPAP vs. standard oxygen, the MD was 0.40 (95% CI: −0.34 to 1.14; *p* = 0.29), and for HFNC and standard oxygen the MD was 0.10 (95% CI: −0.42 to 0.62; *p* = 0.69).

In addition, another analysis was conducted after excluding David et al. (due to a shorter outcome assessment of endpoint timing and high inconsistency) [14]. In this analysis, BiPAP showed a statistically significant benefit over standard oxygen, MD = 2.5 (95% CI: 1.01 to 3.90; *p* < 0.001), and it was the best intervention in 99.9% of simulations. In contrast, HFNC remained not statistically significant compared to standard oxygen, MD = 0.10 (95% CI: −0.41 to 0.62; *p* = 0.69).

### 3.3. Network Meta-Analysis of Observational Studies and Pairwise Meta-Analysis

Seven observational studies (n = 11,200) compared PICU LOS across HFNC, NIV (two studies solely on BiPAP), and standard oxygen [4,15,16,18,20,28,30]. Network structure is included in the Supplementary Data. Reported ages ranged widely from 2 to 9.5 years. Most studies reported moderate to severe asthma exacerbations, except for one multicenter cohort that reported mild disease. Unlike the RCTs, asthma diagnoses were largely derived from hospital coding rather than standardized clinical criteria. This reliance on coding carries a risk of misclassification, particularly in younger children in whom wheezing may reflect alternative underlying etiologies.

**Figure 5:**
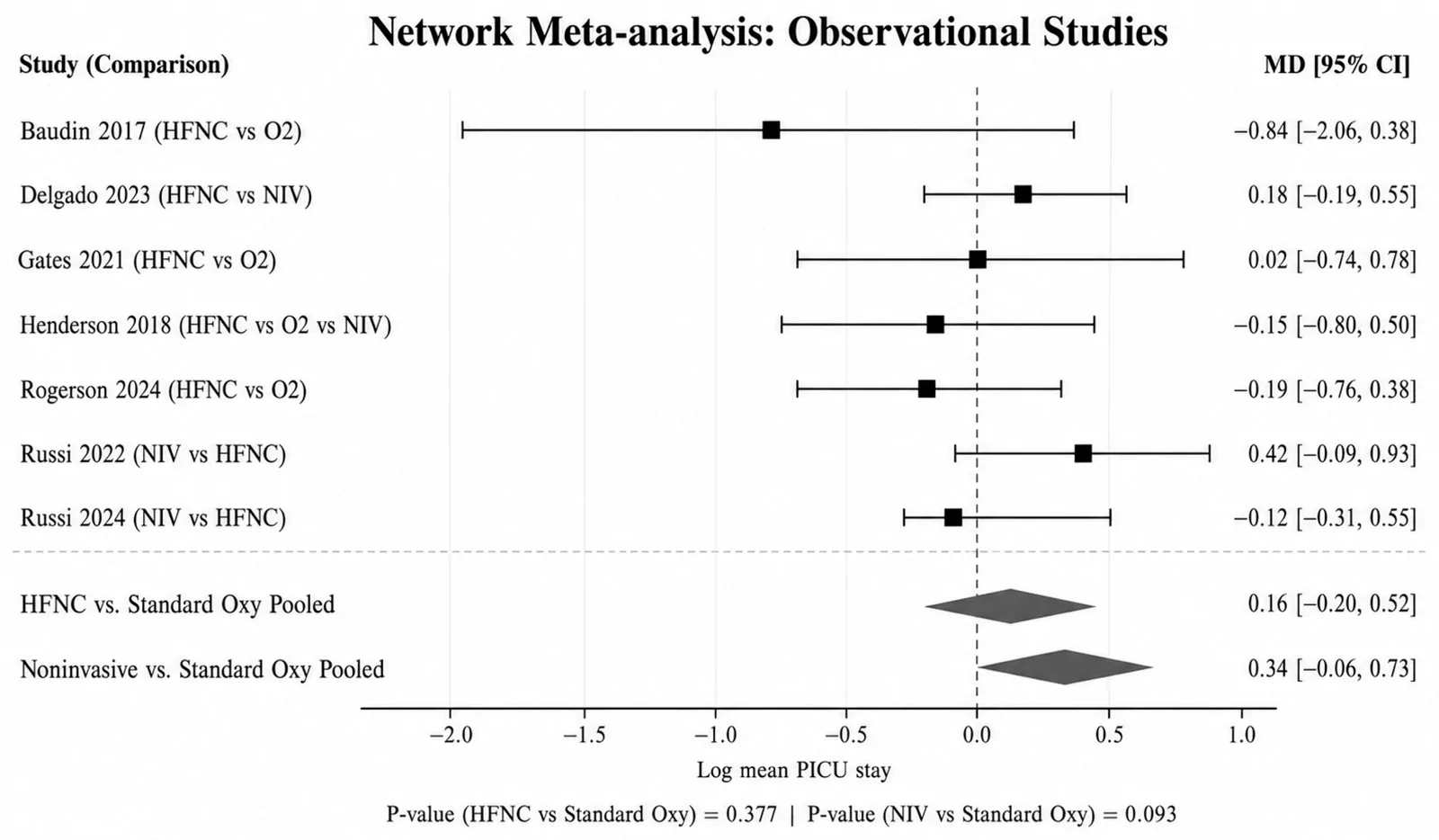
Forest plot of a network meta-analysis of observational studies comparing three modalities: standard oxygen (StandardOxy), high-flow nasal cannula (HFNC), and bilevel positive airway pressure (BiPAP). Squares indicate individual study effect estimates (mean differences), with horizontal lines representing 95% confidence intervals. Rhombuses indicate pooled network meta-analysis estimates, and their widths represent the corresponding 95% confidence intervals [4,15,16,18,20,28,30].

HFNC was associated with a modest increase in PICU LOS compared to standard oxygen, with log MD = 0.16 (95% CI −0.19 to 0.52; *p* = 0.37) and GMR of 1.17 (95% CI: 0.82 to 1.68). NIV showed an increase in PICU LOS compared to standard oxygen with MD = 0.338 (95% CI −0.056 to 0.73; *p* = 0.09) with GMR of 1.4 (95%CI: 0.95 to 2.08), indicating that for both modalities the PICU stay was prolonged; however, this effect was not statistically significant. The forest plot is shown in Figure 5.

BiPAP was non-significantly related to a longer PICU LOS compared to HFNC (mixed comparison incorporating both direct and indirect evidence as described in the statistical analysis) with log MD = 0.18 (95% CI −0.04 to 0.40; *p* = 0.11) with GMR of 1.19 (95% CI: 0.95 to 1.49).

The probabilities of treatment rankings suggested that NIV had the highest probability of a longer PICU LOS, whereas standard oxygen had the lowest likelihood (shorter PICU LOS). Study heterogeneity was very low (0.00%). There was no evidence of small study effect as revealed by Egger’s regression test (*p* = 0.42). The symmetry of the funnel plot indicated that there was no significant publication bias. Inconsistency was not statistically significant in this network; therefore, no sensitivity analysis was conducted (*p* = 0.78).

Subgroup Pairwise Meta-Analyses and all funnel plots are detailed in Supplementary Data.

### 3.4. Age-Related Pattern in Ventilation Modality Use

Observational studies demonstrated an association between age and respiratory support modality in pediatric asthma exacerbations. HFNC was more frequently used in younger children (<6) across five studies [5,20,49], although one study found no significant association [17], and another reported significantly higher HFNC failure rates in infants under 12 months [37]. Conversely, older children were consistently more likely to receive NIV, with five studies finding that children over 6 years had a significantly higher likelihood of NIV initiation [4,5,20,29,49]. IMV findings were heterogeneous, with no consistent age-related pattern identified across studies [5,9,20,26,30,32,38,49,50].

### 3.5. Failure Rate in Ventilation Modalities

We defined treatment failure as escalation to a higher level of support (NIV or IMV) or discontinuation due to complications, expressed as a proportion of the initial number treated. BiPAP was the most frequently reported NIV modality. Due to limited CPAP data, only BiPAP failure rates are presented. Among 3455 children who received BiPAP across 11 studies, 91 patients had treatment failure, yielding a 2.63% failure rate [4,10,21,22,23,24,26,27,28,29,30]. Of 9107 children treated with HFNC in 11 studies, 1149 had treatment failure, yielding a 12.6% failure rate [4,13,14,15,18,19,20,21,28,30,37], significantly higher with HFNC than with BiPAP (OR = 5.32, 95% CI: 4.28–6.68; *p* < 0.001). It should be noted that this result may reflect confounding by indication, unadjusted individual escalation thresholds and center-level practices. Detailed failure rate data are presented in the Supplementary Data.

### 3.6. Mortality Rates Among Ventilation Modalities

Across 15 studies of IMV, 332 out of 6360 patients died, corresponding to a mortality rate of 5.2% [5,9,22,31,33,34,36,38,39,42,46,47,48,49,51]. In the 11 NIV studies, only 2 deaths occurred among 5042 patients, corresponding to a mortality rate of 0.04% [4,21,22,23,27,28,29,30,47,48,49]. Seven HFNC studies recorded 67 deaths among 8694 patients, corresponding to a mortality rate of 0.77% [4,19,21,28,30,47,48].

A pooled comparison across modalities demonstrated significant differences in crude mortality (Pearson Chi^2^ = 503.5; *p* < 0.001; Fisher’s exact *p* < 0.001), with IMV associated with the highest crude rate mortality.

It is important to acknowledge the potential confounding by indication across all observational analyses included in this study, including the mortality analysis, treatment failure analysis and the observational network meta-analysis. Patients receiving IMV are inherently those with the greatest disease severity, making confound by indication an expected source of bias, the available study data did not permit adjustment for baseline disease severity. As elaborated more in the Discussion section.

Heart rate, respiratory rate and adverse events across ventilation are added in the Supplementary File.

### 3.7. Racial and Ethnic Disparities in Ventilation Modalities

(a) Mechanical ventilation:

Two studies found that Black children had higher odds of receiving IMV compared to White children. However, PICU stay and post-intubation ventilation duration did not differ significantly by race. Hispanic children were generally less likely to be intubated, although this was not consistently significant [9,32]. One study reported lower odds of IMV in Hispanic vs. White children, but with no significant difference between Black and White children [50]. Another study reported shorter IMV duration in Black children and longer duration in Hispanic children [39].

(b) Non-invasive mechanical ventilation:

One study found that NIV use among Black children compared to Hispanic children was slightly higher, though the difference was not statistically significant [45]. Another study also reported greater NIV use among Black children relative to HFNC, lower use of NIV in Hispanic/Latino, and no significant difference between modality use for White children [4]. Another study reported a significant increase in noninvasive modalities (NIV and HFNC) use over time across all racial/ethnic groups, with the steepest increase among Hispanic children [5].

(c) High-Flow Nasal Cannula:

Three studies found no significant association between HFNC use and race/ethnicity [19,20,37]. Conversely, one study reported higher HFNC use among Asian and mixed-race children [4]. Another study reported a significant increase in HFNC use over time across all groups, with the steepest increase among Hispanic, Asian/Pacific Islander, and White children [5].

## 4. Discussion

In this systematic review and meta-analysis, we synthesized data on respiratory support modalities for acute asthma exacerbations in children between 2010 and 2025. We found an increase in respiratory support use, with HFNC used the most. HFNC did not demonstrate a clear clinical benefit and was associated with increased PICU length of stay compared to standard therapy and a higher failure rate than NIV. Conversely, NIV improved asthma scores in the emergency department, with low complication and failure rates.

Our findings align with literature supporting BiPAP efficacy in asthma exacerbation. A meta-analysis of adult exacerbations found NIV was associated with faster clinical improvement and lower intubation rates [6]. A 2024 Cochrane review of 3 pediatric asthma RCTs comparing BiPAP to standard therapy found a reduction in intubation rates and shorter PICU stay in the BiPAPgroup [66]. Physiologically, BiPAP improves tidal volume, reduces respiratory rate, and may provide a bronchodilatory effect, making it suitable for severe asthma exacerbations [67]. In contrast, HFNC reduces work of breathing via dead space washout and flow effects but lacks adjustable inspiratory pressure, which may limit its utility in severe exacerbations. A meta-analysis and review in adults with asthma likewise found no clear clinical benefit to HFNC use [68,69].

Our findings may help guide clinical decision-making. While BiPAP ranked as the most effective modality, it is also more cumbersome, often necessitating sedation in severe cases with dyspnea or air hunger. Nevertheless, we recommend considering BiPAP whenever intubation might otherwise be required, given its potential to prevent intubation-related complications.

Conversely, HFNC was associated with longer PICU stay and a higher failure rate, warranting caution regarding its use as a universal escalation strategy in asthma. These findings should be interrupted cautiously because the observational studies were susceptible to confounding by indication meaning patients receiving HFNC could have grater baseline disease severity than those managed with standard oxygen, and adequate adjustment for baseline severity, age and comorbidities was not possible.

HFNC was used more frequently in younger children (<6) in observational studies. This may reflect real-world practice preferences driven by diagnostic uncertainty, where distinguishing asthma from bronchiolitis or viral-induced wheeze remains challenging. Given HFNC efficacy in bronchiolitis, clinicians may therefore extend HFNC use to children with undifferentiated wheeze, independent of a confirmed asthma diagnosis [68]. Conversely, NIV was more prevalent in older children (>6), in whom asthma diagnosis is more certain and the pathophysiological rationale for NIV is better established. This pattern suggests hospital escalation strategies may not be strictly asthma-specific and may therefore not represent truly effective asthma therapy.

Our analysis also highlights racial and ethnic disparities. Black children more frequently received invasive ventilation, likely reflecting more severe exacerbations. These worse outcomes may also reflect broader socioeconomic and environmental challenges, such as greater allergen exposure and reduced access to specialty care [70]. Similarly, Hispanic children had increased use of NIV and HFNC, possibly due to reduced access to primary care and language barriers, underscoring the need for tailored preventive interventions [71,72]. For example, an RCT providing culturally sensitive education to Puerto Rican families demonstrated improved asthma control and reduced emergency department visits and hospitalizations [73].

Our work has several important limitations. The studies were methodologically heterogeneous, including retrospective and prospective cohorts, with only five RCTs. Many studies had missing data, particularly regarding asthma scores, disease severity, ventilation parameters, and adverse events. Observational studies were subject to confounding by indication. Furthermore, five key studies introduced inconsistency into the network, limiting the robustness of our conclusions. Finally, racial and ethnic data were inconsistently reported, limiting our ability to systematically evaluate disparities in care.

## 5. Conclusions

In conclusion, our network meta-analysis provides a clinically plausible signal that NIV (particularly BiPAP) may offer greater short-term improvement in asthma exacerbation, while HFNC has not demonstrated a clear advantage over standard therapy. Given the low certainty of the evidence, our findings should be interpreted with caution. Future adequately powered RCTs using pre-specified standardized scores, explicit bronchiolitis exclusion, and age-stratified analysis are essential to clarify optimal ventilation strategies in children with moderate to severe asthma.

## 6. Patents

This study is a systematic review and meta-analysis with no patents included.

## Figures and Tables

**Table 1:** Summary of randomized controlled trials evaluating respiratory support in pediatric patients with acute asthma exacerbation.

Authors and Year	Study Design	Median Age Range	Total Participants	Intervention	Control Group	Primary Outcome	Secondary Outcome
**Basnet et al., 2012 [10]**	Pilot CT	6 1–18	**38** BiPAP 18 Control 20	**BiPAP** Noninvasive positive pressure ventilation initiated early via BiPAP mask, with initial settings of inspiratory pressure 8 cm H_2_O and expiratory pressure 5 cm H_2_O, titrated to achieve tidal volume of 6–9 mL/kg. Albuterol was administered via nebulizer through the BiPAP circuit.	**Standard therapy** Continuous nebulized albuterol (0.5 mg/kg; max 15 mg/h), IV methylprednisolone 2 mg/kg/day (max 80 mg/day), and supplemental oxygen via nasal cannula or face mask to maintain SpO_2_ > 92%.	Asthma score improvement CAS	Respiratory rate improvement
**Ballestero et al., 2018 [11]**	Pilot CT	3 1–14	**60** HFNC 30 Control 30	**HFNC** Heated, humidified oxygen via HFNC system with flows adjusted to 2 L/kg/min and FiO_2_ titrated to maintain SpO_2_ > 93%. Standard asthma treatment continued as needed (e.g., corticosteroids, bronchodilators).	**Standard oxygen therapy** Nasal prongs, Venturi mask, or non-rebreather mask. Both groups received the same pharmacologic asthma protocol.	Asthma score improvement PS	PICU length of stay, additional therapies
**Gauto Benitez et al., 2019 [12]**	RCT	5.4 * 2–14	**119** HFNC 57 Control 62	**HFNC** Flow starting at 1 L/kg/min and titrated up to 2 L/kg/min to achieve SpO_2_ 93–98%. All patients received continuous salbutamol nebulization (10–20 mg/h), IV dexamethasone 0.6 mg/kg (max 12 mg), and IV magnesium sulfate (200 mg/kg over 4 h).	**Standard oxygen therapy** Nasal cannula, simple mask, or non-rebreather mask, titrated to the same SpO_2_ goal. Identical pharmacologic regimen was used in both groups.	Asthma score improvement PIS	Length of ED stay, PICU admission
**Kapur et al., 2024 [13]**	Feasibility RCT	5 (mean) 2–11	**28** HFNC 14 Control 14	**HFNC** Flow at 2 L/kg/min for first 10 kg, plus 0.5 L/kg/min for each kg beyond that (max 40 L/min). Bronchodilators delivered using nebulizer; FiO_2_ adjusted to maintain SpO_2_ ≥ 92%.	**Standard oxygen therapy** bronchodilators, systemic corticosteroids. Oxygen administered by standard nasal cannula or mask if needed.	Treatment escalation (failure)	Hospital length of stay
**David et al., 2024 [14]**	RCT	7.5 (mean) 5–16	**50** HFNC 25 BiPAP 25	**HFNC** Continuous high-flow oxygen therapy adjusted to weight: 2 L/kg/min for first 10 kg + 0.5 L/kg/min for each kg above that. Administered via nasal cannula with humidification and FiO_2_ adjusted as needed. Sessions also included 3 sets of 10 diaphragmatic breathing exercises per day.	**BiPAP** Bilevel PAP via mask for 45 min/day with IPAP 12 cm H_2_O and EPAP 8 cm H_2_O, adjusted to improve tidal volume and comfort. Same respiratory exercises were given. Both groups received standard pharmacologic asthma care including salbutamol and corticosteroids.	Hospital length of stay	Asthma score improvement (PASS, PAS, PIS)

* 5 years in HFNC group, 4 years in Control group. HFNC, high-flow nasal cannula; BiPAP, bilevel positive airway pressure; ED, emergency department; PICU, pediatric intensive care unit; CAS, Clinical Asthma Score; PS, Pulmonary Score; PIS, Pulmonary Index Score; PAS, pediatric asthma score; PASS, pediatric asthma severity score; **PRAM**, pediatric respiratory assessment measure; RCT, randomized controlled trial; CT, clinical trial; **FiO_2_**, fraction of inspired oxygen; SpO_2_, peripheral oxygen saturation; **IPAP**, Inspiratory Positive Airway Pressure; **EPAP**, Expiratory Positive Airway Pressure.

**Table 2:** Summary of Observational Studies Evaluating Respiratory Support in Pediatric Asthma.

Authors and Year	Study Design	Number of Participants	Median Age Range	Exposure/Control	Primary Outcome	Key Conclusions *
**Baudin et al., 2017 [15]**	Retrospective Cohort	69 (HFNC 39, standard O_2_ 30)	3.6 1–18	HFNC vs. standard Oxygen	Clinical improvement, HFNC failure rate and PICU LOS	HFNC significantly clinical improvement within 24 h. %5 failures, PICU LOS was longer in HFNC group.
**Henderson et al., 2018 [16]**	Retrospective Cohort	262 (standard 184, HFNC 26, NIV 37, IMV 15)	6 2–17	HFNC/NIV/IMV/ Standard oxygen.	PICU length of stay.	HFNC and NIV had similar PICU LOS. IMV had the longest PICU stay
**Gonzalez Martinez et al., 2019 [17]**	Retrospective Cohort	536 (HFNC 40, standard oxygen 496)	5 4–15	HFNC vs. standard oxygen	PICU admission and clinical improvement	HFNC led to significant clinical improvement. Higher PICU transfer in HFNC group 25%
**Gates et al., 2021 [18]**	Retrospective Cohort	171 (HFNC 104, aerosol mask 67)	HFNC 5, control 7 2–17	HFNC vs. aerosol mask	Hospital, PICU length of stay and clinical improvement	HFNC and aerosol mask had similar hospital LOS, PICU LOS, and MPIS improvement. HFNC had shorter albuterol duration.
**Rogesrson et al., 2023 [19]**	Retrospective Cohort Matched	1766 matched pairs	— 2–18	HFNC vs. standard oxygen	Hospital length of stay	HFNC use was associated with significantly longer hospital and higher NIV use 21.3%.
**Rogerson et al., 2024 [20]**	Retrospective Cohort Matched	443 matched pairs	HFNC 6, control 8 2–18	HFNC vs. oxygen facemask	Clinical improvement, PICU and hospital length of stay	HFNC group slower clinical improvements. Also had longer PICU and hospital LOS.
**Russi et al., 2024 [4]**	Retrospective Cohort Multicentric	10,083	HFNC 6.9 (mean) NIV 8.86 (mean) 2–17	HFNC vs. NIV (CPAP and BiPAP)	Ventilation trends, HFNC and NIV failure.	HFNC use increasing; NIV decreasing. HFNC had higher failure but lower intubation than NIV.
**Pilar et al., 2017 [21]**	Retrospective Cohort	42 (HFNC 20, NIV 22)	HFNC 2.98 NIV 3.74 1.5–14	HFNC vs. NIV	PICU length of stay and treatment escalation.	HFNC failure 40%, 0% BiPAP failure, HFNC was associated with longer PICU stay.
**Abramo et al., 2017 [22]**	Retrospective Cohort	1430 (BiPAP 325, Non-BiPAP 1105) **	4 0.80–18.25	BiPAP vs. Non BiPAP	Asthma score improvement, PICU admission and length of stay	BiPAP well tolerated with significant clinically improvement and BiPAP reduced PICU admission and stay
**Usala and Wilson 2022 [23]**	Retrospective Cohort	101 (NIV 54, Non-Niv 47)	7 (mean) 1–21	NIV vs. Non-NIV	PICU length of stay and adverse events	PICU LOS was longer in the BiPAP group than CPAP or non-NIV. NIV was safe and well tolerated with no major adverse events.
**Mayordomo-Colunga et al., 2011 [24]**	Prospective observational study	72 admissions ***	3.2 —	NIV only	Clinical improvement, need for intubation.	NIV was associated with significant clinical improvement, 5 patients required intubation.
**Authors and year**	**Study design**	**Number of participants**	**Median age** **Range**	**Exposure/Control**	**Primary Outcome**	Key conclusions **
**Dantas Gomes et al., 2013 [25]**	Prospective cross sectional	19	5–12	CPAP only	Clinical improvement asthma severity score	clinical and score (PI) improvement.
**Williams et al., 2011 [26]**	Retrospective and Prospective cohort	165	3.7 (mean) 0.6–8.3	BiPAP only	BiPAP safety, Asthma score improvement, and intubation rate.	Improvement in PAS score, good tolerance and 2.4% intubation rate.
**Kang et al., 2020 [27]**	Retrospective Cohort	46 admissions *** (25 BiPAP, 21 Non-BiPAP)	4.3 (mean) <18	BiPAP vs. standard oxygen	Clinical improvement PICU and Hospital length of stay	BiPAP group showed significant clinical improvement BiPAP longer PICU and hospital stay.
**Delgado et al., 2023 [28]**	Prospective observational study	76 (HFNC 56, NIV 6, combined 14)	HFNC 2.4, NIV 1.16 0.3–13.3	HFNC vs. NIV	PICU length of stay and escalation	HFNC group had shorter PICU stay. No patient was escalated to NIV
**Al-Eyadhy et al., 2015 [29]**	Retrospective Cohort–Comparison two cohorts	124 (non-BiPAP 100, BiPAP 24)	3.6 (mean- 2003) 5.5 (mean -2013)	BiPAP vs. non-BiPAP	NIV trend, PICU length of stay, escalation.	Increase use of NIV, intubation rate 0%, higher PICU length of stay in 2013 cohort study.
**Authors and year**	**Study design**	**Number of participants**	**Median age** **Range**	**Exposure/Control**	**Primary Outcome**	Key conclusions **
**Russi et al., 2022 [30]**	Retrospective Cohort	39 (BiPAP 26, HFNC 13)	BiPAP 10.9 (mean) HFNC 6.8 (mean) 5–17	HFNC vs. BiPAP	PICU and hospital stay, intubation rate	BiPAP group had slightly longer PICU stays, it was not a significant difference. Both groups had similar total hospital stays. Intubation rate was 0%
**Deho et al., 2010 [31]**	Retrospective study	60 (IMV 51, Non-IMV 9)	7.2 (mean) 2–15.5	IMV vs. non-IMV	Complications during and after intubation	70.5% from intubated patients experienced complications. Mortality 0%.
**Sala et al., 2012 [32]**	Retrospective Cohort Multicentric	3318 (IMV 201, Non-IMV 3117)	6.75 (mean) <18	IMV vs. non-IMV	PICU length of stay, asthma mortality score.	IMV was associated with longer PICU stay of length and worse severity scores.
**Hon et al., 2010 [33]**	Retrospective Cohort	30 admissions *** (IMV 6, Non-IMV 24)	3.1 —	IMV vs. non-IMV	PICU length of stay and intubation rate	IMV was associated with longer PICU, 20% intubation rate.
**Sheikh et al., 2013 [34]**	Retrospective Cohort	222 admissions *** (IMV 17, Non-IMV 205)	11 (mean) 5–20	IMV vs. non-IMV	PICU and hospital length of stay, intubation rate	IMV was associated with longer hospital and PICU stay, 8% intubation rate.
**Authors and year**	**Study design**	**Number of participants**	**Median age** **Range**	**Exposure/Control**	**Primary Outcome**	Key conclusions **
**Boeschoten et al., 2018 [35]**	Retrospective Cohort Multicenter	660 admissions *** (IMV 118, Non-IMV 542)	— 2–18	IMV vs. non-IMV	Ventilation trends, and length of stay	IMV was associated with longer PICU, IMV decrease while HFNC use increased.
**Cheng et al., 2020 [36]**	Retrospective Cohort	67 admissions *** (IMV 7, non-IMV 60)	3.3 —	IMV vs. non-IMV	PICU length of stay and ventilation trends	IMV was associated with longer PICU and hospital stay, increase use of NIV and HFNC. NIV did not reduce intubation rate
**Dunbar et al., 2024 [37]**	Retrospective Cohort	195 (51 asthmatic patients on HFNC)	1.4 0–18	HFNC	HFNC failure	Asthmatic patients are associated with 39% increased risk of HFNC failure
**Shibata et al., 2014 [9]**	Retrospective Cohort	157 (intubated)	— <18	IMV	Intubation at community and tertiary ER, race comparison	Children from community ERs were more likely to be intubated before PICU admission. African American children had a significantly higher likelihood of being intubated prior to PICU admission and even after justification for age, gender, PIM2 score.
**Rampa et al., 2015 [38]**	Retrospective observational	250,718	≤21	IMV	Ventilation trends (IMV), mortality and hospital stay	IMV needed in 0.55% of cases; mortality was 4% for ventilated patients vs. 0.03% overall. IMV associated with significantly higher length of stay.
**Newth et al., 2012 [39]**	Retrospective Cohort Multicenter	261	6.8 1–18	IMV	Duration of ventilation, PICU outcome and mortality	Median ventilation 42 h. African-Americans overrepresented. Shorter duration of MV and PICU LOS if intubated before PICU admission. Mortality 4.2%
**de Miguel-Diez et al. 2014 [40]**	Retrospective Epidemiological Study	12,038	5.79 (mean) 0–15	IMV, NIV, HFNC	Trends in ventilation use and hospitalization	Decreased hospitalizations for pediatric asthma. Increased Use of NIV. IMV remained stable.
**Rogerson CM. et al., 2024 [41]**	Retrospective Cohort Multicentric	77,115	>2	IMV, NIV, HFNC	Ventilation Trends	Decrease in IMV usage, increase in both HFNC and NIV.
**Authors and year**	**Study design**	**Number of participants**	**Median age** **Range**	**Exposure/Control**	**Primary Outcome**	Key conclusions **
**Hartman et al. 2010 [42]**	Retrospective Cohort	28,309 admissions ***	5	IMV, NIV, HFNC	Ventilation trends and mortality	mechanical ventilation remained low. Mortality rate remained low.
**Gutierrez-Albaladejo et al. 2023 [43]**	Retrospective Cohort	85,664	— 0–15	IMV, NIV, HFNC	Trends in hospital admissions, mechanical ventilation use and in-hospital mortality (IHM)	Hospital admissions for asthma significantly declined. Use of NIV doubled. IMV remained rare (<1%). mortality among hospitalized children with asthma decreased by more than half over the study period
**Smith et al. 2020 [44]**	Cross-sectional study	95,204	6 2–17	IMV, NIV, HFNC	Ventilation trends, ICU admission, length of stay, and mortality	NIV use increased, IMV remained stable. ICU admissions were higher in high-NIV centers. IMV rates, mortality, and LOS were unaffected by NIV trends.
**Lee et al. 2020 [45]**	Retrospective Cohort	925	7.5 (mean) 2–18	IMV, NIV, HFNC	Race comparison in ventilation usage.	African American more ICU admissions, no difference in NIV usage between Hispanic and African American.
**Mukherjee et al., 2022 [46]**	Prospective cohort	2195	0–14	IMV, NIV, HFNC	Ventilation trends and mortality	Most IMV patients were (0–4 years). Mortality in PICU was 0.7%. No significant trend was found overtime in the usages of ventilation.
**Salie et al., 2025 [47]**	Retrospective Audit	180	5.6 0–16	IMV, NIV, HFNC	Ventilation trends and mortality	HFNC increase use over time especially after 2013. IMV remained low. No major shift in NIV use.
**Monteverde-Fernandez et al., 2021 [48]**	Cross sectional Multicenter	434	— 2–14	IMV, NIV, HFNC	Variability in respiratory support management	Wide variability in respiratory support and adjunct therapies across Latin America; IMV used in 6%.
**Craig et al., 2022 [49]**	Retrospective Cohort Multicenter	14,029	3 1–17	IMV, NIV, HFNC	Frequency and pattern of respiratory support.	HFNC was most used respiratory support. Very low use of NIV modality.
**Authors and year**	**Study design**	**Number of participants**	**Median age** **Range**	**Exposure/Control**	**Primary Outcome**	Key conclusions **
**Hasegawa et al. 2013 [50]**	Retrospective Cross sectional	592,805	0–17	IMV, NIV, HFNC	Ventilation and hospitalization trends and mortality.	Decrease in pediatric asthma hospitalizations and in-hospital mortality. Mechanical ventilation slightly increased and NIV significantly increased.
**Smith MA et al. 2023 [5]**	Retrospective Cohort Multicenter	67,614	2–18	IMV, NIV, HFNC	Ventilation trends and mortality	IMV use decrease to half. NIV use more than doubled. HFNC use also significantly increased. Mortality remained low (0.35%), almost all among intubated patients.
**Rampersad et al. 2018 [51]**	Retrospective Cohort	589 admissions ***	4.7 —	IMV, NIV, HFNC	Ventilation trends and mortality.	Increased use of both NIV and IMV increased. NIV did not reduce IMV. Mortality was low (0.6%).

* Key conclusions are reported as stated by the authors of the original articles. ** Number in PICU. *** Refers to number of **admissions**, not number of patients. BiPAP, Bilevel Positive Airway Pressure; CPAP, Continuous Positive Airway Pressure; HFNC, High-Flow Nasal Cannula; IMV, Invasive Mechanical Ventilation; NIV, Non-Invasive Ventilation; PICU, Pediatric Intensive Care Unit; LOS Length of Stay; PAS, Pediatric Asthma Severity; PI, Pulmonary Index Score; MPIS, Modified Pulmonary Index Score; PIM2, Pediatric Index of Mortality 2.

## Data Availability

Anonymized data will be provided upon reasonable request to the corresponding author.

## Supplementary Materials

The following supporting information can be downloaded at: https://www.mdpi.com/article/10.3390/pediatric18040104/s1, Table S1. Demographic Data (Country, Gender, Race/Ethnicity) in RCT Articles; Table S2. Demographic Data (Country, Gender, Race/Ethnicity) in Observational Studies; Table S3. Certainty of Evidence Ratings for Observational Studies (GRADE Framework); Table S4. Trends in Ventilation Modalities Among PICU Patients, 2009–2019; Table S5. Trends in Ventilation Modalities Among Hospitalized Patients, 2009–2019; Table S6a. RCT Network Meta-analysis of RCTs: Ranking Each Treatment for asthma score improvement (Estimated probabilities (%) for each treatment rank, assuming lower asthma scores indicate better outcomes); Table S6b. Network Meta-analysis for observational studies: Ranking of treatments based of PICU length of stay (Estimated probabilities (%) of each treatment rank, assuming shorter PICU stay indicated better outcome); Table S7. Use and Failure Rates of BiPAP in Included Studies; Table S8. High-Flow Nasal Cannula (HFNC) Use and Failure Rates Across HFNC Studies; Table S9. Mortality in Pediatric Asthma Patients Receiving IMV; Table S10. Mortality in Pediatric Asthma Patients Receiving NIV; Table S11. Mortality in Pediatric Asthma Patients Receiving HFNC. Table S12: PRISMA 2020 checklist. Figure S1a. Risk of bias assessment of individual randomized controlled trials using the Cochrane RoB 2.0 tool; Figure S1b. Risk of Bias Summary Plot; Figure S2a. Network meta-analysis map of randomized control trials between 3 modalities: standard oxygen (Standardoxy), high-flow nasal cannula (HFNC), and bilevel positive airway pressure (BiPAP); Figure S2b. Network meta-analysis map of observational studies between 3 modalities: standard oxygen (standardoxy), high-flow nasal cannula (HFNC), and non-invasive ventilation (NIV); Figure S3a. Meta-analysis of PICU Length of Stay (LOS) in HFNC vs. Standard Oxygen Pediatric Asthma Patients; Figure S3b. Meta-analysis of PICU Length of Stay (LOS) in HFNC vs. NIV Pediatric Asthma Patients; Figure S3c. Meta-analysis of PICU Length of Stay (LOS) in Intubated vs. Non-intubated Pediatric Asthma Patients; Figure S4a. Funnel plot of network randomized control trials. Egger’s bias *p* = 0.53; Figure S4b. Funnel plot of network observational studies. Egger’s bias *p* = 0.09; Figure S4c. Funnel plot, intubated versus non-intubated. Egger’s bias *p* = 0.5; Figure S4d. Funnel plot, HFNC versus standard oxygen, Egger’s bias *p* = 0.068; Figure S4e. Funnel plot, HFNC versus NIV. Egger’s bias *p* = 0.75.

## Abbreviations

The following abbreviations are used in this manuscript:

BiPAP	Bilevel Positive Airway Pressure
CAS	Clinical Asthma Score
CI	Confidence Interval
CPAP	Continuous Positive Airway Pressure
ED	Emergency Department
EPAP	Expiratory Positive Airway Pressure
GRADE	Grading of Recommendations, Assessment, Development and Evaluations
GMR	Geometric Mean Ratio
HFNC	High-Flow Nasal Cannula
HR	Heart Rate
IMV	Invasive Mechanical Ventilation
IQR	Interquartile Range
LOS	Length of Stay
MD	Mean Difference
NIV	Non-Invasive Ventilation
NMA	Network Meta-Analysis
OR	Odds Ratio
PICU	Pediatric Intensive Care Unit
PIS	Pulmonary Index Score
PS	Pulmonary Score
PRISMA	Preferred Reporting Items for Systematic Reviews and Meta-Analyses
RCT	Randomized Controlled Trial
REML	Restricted Maximum
