# Supplementary material for: Comparing Respiratory Support Modalities in Pediatric Asthma Exacerbation–A Systematic Review and Meta-Analysis
Source: Pediatr Rep. 2026 Aug 4;18(4):104. doi: 10.3390/pediatric18040104 (PMC13516637; doi:10.3390/pediatric18040104)
Supplement: Supplementary file 1 [file pediatrrep-18-00104-s001.zip › pediatrrep-4457974-supplementary.pdf]

Online Supplement:

Search Strategy:

A comprehensive search strategy was developed for each database using both Medical Subject Headings (MeSH) and relevant keywords. Filters were applied only in PubMed to limit results to studies involving humans, children, and publications in English. Additionally, all database searches were restricted to articles published from January 1, 2010, onwards. No restrictions were applied regarding study design.

Date of the search: 17/3/25.

PubMed:

("Child"[Mesh] OR pediatric OR child OR infant OR adolescent) AND (("Asthma"[Mesh] OR asthma (tiab) OR asthma OR status asthmaticus) AND (("Respiration, Artificial"[Mesh] OR "Mechanical Ventilation"[Mesh] OR "Positive-Pressure Respiration"[Mesh] OR CPAP OR BIPAP OR "mechanical ventilation" OR "noninvasive ventilation" OR "high-flow nasal cannula" OR Respiratory support).

Cochrane:

(mh Child OR pediatric OR child OR infant OR adolescent)AND (mh Asthma OR asthma (tiab) OR asthma OR "status asthmaticus") AND (mh "Respiration, Artificial" OR mh "Mechanical Ventilation" OR mh "Positive-Pressure Respiration" OR CPAP OR BIPAP OR "mechanical ventilation" OR "noninvasive ventilation" OR "high-flow nasal cannula" OR "Respiratory support")

Scopus:

(((child OR pediatric OR infant OR adolescent) AND ((asthma OR "status asthmaticus") AND (ventilation OR "mechanical ventilation" OR "noninvasive ventilation" OR "high-flow nasal cannula" OR "respiratory support" OR "positive-pressure respiration"))) AND PUBYEAR > 2009

Embase:

(child OR pediatric OR infant OR adolescent) AND (asthma OR 'status asthmaticus') AND (ventilation OR 'mechanical ventilation' OR 'noninvasive ventilation' OR 'high-flow nasal cannula' OR 'respiratory support' OR 'positive-pressure respiration') AND [2010-2025]/py

Keyword: HFNC, NIV, Asthma exacerbations, Invasive ventilation.

Authors and year	Country	Gender	Race and ethnicity
**Basnet et al.,2012 [14]**	**USA**<br>**(Illinois)**	**NPPV 40%**<br>**Control 70%**	**— — — –**
**Ballestero et al., 2018 [19]**	**Spain**	**HFNC 53%**<br>**Control 56%**	**— — — –**
**Gauto Benitez et al.,2019 [26]**	**Paraguay**	**HFNC 31.3%**<br>**Control 48.5%**	**— — — –**
**Kapur et al., 2024 [27]**	**United Kingdom**	**HFNC 64.3%**<br>**Control 63.6%**	**White: 77.6%, Black: 8.2%, Asian: 8.2%, Mixed/Other: 6.1%**
**David et al., 2024 [20]**	**Brazil**	**HFNC :60%**<br>**Control: 52%**	**— — — –**

Table S1. Demographic Data (Country, Gender, Race/Ethnicity) in RCT Articles

Authors and year	Country	Gender (% male)	Race and ethnicity
Baudin et al., 2017 [1]	France	HFNC: 51.3%<br><br>Control: 70%	– — — — –
Henderson et al., 2018 [2]	USA (Utah)	56%	72% White, 18% Hispanic, 3% African American, and 7% other
Gonzalez Martinez et al., 2019 [28]	Spain	HFNC 67.5%<br><br>Control 63.1%	– — — — –
Gates et al., 2021 [3]	USA (North Carolina)	HFNC: 49%<br><br>Aerosol Mask: 57%	– — — — –
Rogesrson et al., 2023 [29]	USA (Indiana)	HFNC 57.7%<br><br>Control 58.6%	HFNC: 58% White, 37% Black, 9.8% Hispanic; Controls: 57% White, 37.6% Black, 9.7% Hispanic.
Rogerson et al., 2024 [4]	USA (Indiana)	HFNC 57.3%<br><br>Control 58.2%	HFNC group: 48.3% Black, 15.1% Hispanic; Control group: 53.7% Black, 12.9% Hispanic
Kang et al., 2020 [17]	Taiwan	BiPAP 28%, Non-BiPAP 57%	– — — — –

Authors and year	Country	Gender (% male)	Race and ethnicity
**Russi et al., 2024 [23]**	**USA (125 centers)**	**HFNC 57.3%**<br>**BiPAP 56.1%**<br>**CPAP 59.1%**	**33.7% Black, 24.9% White, 15% Hispanic/Latino, 3.5% Asian, 0.6% Native American/Alaskan, 0.4% Hawaiian/Pacific Islander, 5.4% Mixed/Other, and 16.7% unknown**
**Pilar et al., 2017 [30]**	**Spain**	**HFNC 60%**<br>**NIV 77%**	– — — — –
**Abramo et al., 2017 [15]**	**USA (Tennessee)**	**59.8%**	– — — — –
**Usala and Wilson 2022 [31]**	**USA (New York)**	**BiPAP 46%**<br>**CPAP 50%**<br>**Non-NIV 47%**	**BPAP: 78.3% Hispanic, 17.4% Black, 0% White.**<br>CPAP**: 57% Hispanic, 27% Black, 13% White.**<br>Non-NIV**: 60% Hispanic, 28% Black, 6% White**
**Mayordomo-Colunga et al., 2011 [16]**	**Spain**	**69.4%**	– — — — –
**Gomes et al., 2013 [13]**	**Brazil**	**63%**	– — — — –
**Williams et al., 2011 [25]**	**USA (Tennessee)**	**64%**	– — — — –
**Delgado et al., 2023 [6]**	**Spain**	**HFNC 54% , NIME 33%**	– — — — –

Authors and year	Country	Gender (% male)	Race and ethnicity
Al-Eyadhy et al., 2015 [32]	Saudi Arabia	44.8%	– — — — –
Russi et al., 2022 [7]	USA (Florida)	HFNC 46%<br><br>BiPAP 46%	– — — — –
Deho et al., 2010 [33]	England	58.3%	– — — — –
Sala et al., 2012 [8]	USA (multi-center)	Intubated 58%<br><br>Non-intubated 61%	Intubated 49% African American, 32% White, 12% Hispanic, 8% Other. Non-Intubated: 41% African American, 34% White, 15% Hispanic, 10% Other
Craig et al., 2022 [34]	Australia and New Zealand	62.9%	– — — — — —
Hon et al., 2010 [9]	Hong Kong	Intubated 83%<br><br>Non-intubated 40%	– — — — –

Authors and year	Country	Gender (% male)	Race and ethnicity
Sheikh et al., 2013 [10]	USA (Ohio)	Intubated 59%<br><br>Non-intubated 58%	– — — — –
Boeschoten et al., 2018 [11]	Netherlands	Intubated 67%<br><br>Non-intubated 57%	overall: 64% White, 36% non-White
Cheng et al., 2020 [12]	Hong Kong	Intubated 43%<br><br>Non-intubated 53%	– — — — –
Dunbar et al., 2024 [35]	USA (Colorado)	47% (Overall not specific to asthma)	80% White, 21% non-White/other
Shibata et al., 2014 [36]	USA (multi-center)	60%	40.8% White, 33.8% Black, 25.5% Other
Rampa et al., 2015 [37]	USA (multi-state)	61.7%	35% White, 32% Black, 23.2% Hispanic, 6.4% Other, 2.6% Asian/Pacific Islander, 0.9% Native American.
Newth et al., 2012 [21]	USA (multi-center)	62%	62% African American, 33% White, 3% Asian, 2% Other

Authors and year	Country	Gender (% male)	Race and ethnicity
**Miguel-Diez et al. 2014 [38]**	**Spain**	**In 2010 63.5%**	**– — — — — –**
**M. Rogerson et al., 2024 [4]**	**USA (multi-state)**	**59.1%**	**38.% Black, 26.4% White, 35.6% Other**
**Gutierrez-Albaladejo et al. 2023 [39]**	**Spain**	**In 2020 59%**	**– — — — — –**
**Alla Smith et al. 2020 [40]**	**USA**	**60.6%**	**42.4% Black, 36.4% White, 2.8% Asian, 18.4% Other/Unknown**
**S. Lee et al. 2020 [41]**	**USA (New York)**	**Hispanic 57%**<br><br>**African American 54%**	**64% Hispanic**<br><br>**36% African American.**
**Mukherji et al.,2022 [42]**	**England**	**58.6%**	**– — — — — –**
**Salie et al., 2025 [43]**	**South Africa**	**53.3%**	**– — — — — –**
**Monteverde-Fernandez et al., 2021 [44]**	**Latin American countries (Argentina, Brazil, Chile, Colombia, Ecuador, Mexico, and Uruguay)**	**47%**	**68% Caucasian, 31% Mestizo, 1% African American or Indigenous**

Authors and year	Country	Gender (% male)	Race and ethnicity
Hasegawa et al. (2013) [45]	USA (multi-state)	In 2009 62.9%	In 2009: 31% White, 28.5% Black, 19% Hispanic, 7.7% Other
Smith et al. (2023) [5]	USA (multi-center)	59.5%	25.1% White, 38.5% Black, 12.9% Hispanic, 2.4% Asian/Pacific Islander, 4.6% Other, 16.6% Unknown
Rampersad et al. (2018) [24]	Australia	57.7%	– — — — — –
Hartman et al. 2010 [22]	USA (New Jersey)	59.6%	35.2% White, 38.4% Black, 17.4% Hispanic, 9.1% Other

Table S2. Demographic Data (Country, Gender, Race/Ethnicity) in Observational Studies.

GRADE assessment table

Certainty assessment							Summary of findings
Participants (studies) Follow-up	Risk of bias	Inconsistency	Indirectness	Imprecision	Publication bias	Overall certainty of evidence	
**In-hospital mortality - Respiratory Support Modalities.**							
23 non-randomized studies	not serious [a,b,c]	not serious [d,e,f]	not serious	not serious [g,h,i]	None	⊕⊕◯◯ Low[a,b,c,d,e,f,g,h,i]	
**Ventilation failure rate - NIV (BiPAP) and HFNC**							
15 non-randomized studies	not serious	not serious	not serious	not serious	None	⊕⊕◯◯ Low[j,k,l,m,n,o]	
**Adverse events – Respiratory Support Modalities**							
7 non-randomized studies	not serious [p,q,r]	not serious [s,t,u]	not serious	not serious [v]	None	⊕⊕◯◯ Low [p,q,r,s,t,u,v]	
**PICU length of stay (NIV vs HFNC)**							

GRADE assessment table

Certainty assessment							Summary of findings
4 non-randomized studies	not serious [w]	not serious [x]	not serious	not serious [y]	None	⊕⊕◯◯ Low [w,x,y]	GMR **0.88 days higher** (0.86 higher to 0.9 higher)
**PICU Length of Stay (LOS) in Intubated vs. Non-intubated**							
5 non-randomized studies	not serious [z]	not serious [aa]	not serious	Serious [ab]	None	⊕◯◯◯ Very low [aa,ab,z]	GMR **1.81 day higher** (0.86 higher to 4.6 higher)
**PICU Length of Stay: HFNC vs. Standard Oxygen**							
4 non-randomized studies	not serious [ac]	not serious [ad]	not serious	not serious [ae]	None	⊕⊕◯◯ Low[ac,ad,ae]	GMR **0.41 higher** (0.03 higher to 0.79 higher)

Explanation:

a. IMV: Although 13 of 15 studies were rated as having moderate risk of bias, the largest and most heavily weighted studies (e.g., Smith et al. [5], Craig et al. [34]) were rated as low risk. The moderate-risk studies were generally well conducted, with objective outcome assessment and complete follow-up, and none were rated as high risk. Given the consistency and strength of findings across the evidence base, we judged the risk of bias as not serious.

b. NIV: Majority of studies (8 of 11) had moderate risk of bias, mainly due to retrospective designs, lack of confounder adjustment, and no control groups. Even though mortality is an objective outcome, the absence of adjustment for baseline illness severity (e.g., age, comorbidities, asthma control) introduces confounding by indication. These factors lower confidence in estimating mortality risk even within NIV-treated patients.

c. HFNC; Four of the seven studies were judged to have low risk of bias, including large-scale or well-matched cohorts with complete follow-up and reliable outcome ascertainment. The remaining three studies were moderate risk, primarily due to confounding or retrospective design, but all used hospital records to measure mortality, an objective outcome unlikely to be misclassified. Therefore, the risk of bias is not considered serious.

d. IMV: Although reported mortality rates ranged from 0% to 7.5%, the direction of effect was consistent across studies, with IMV consistently associated with higher mortality. Variability was explainable by differences in study design, illness severity, and sample size. No unexplained heterogeneity was identified.

e. NIV: Mortality was consistently 0% across 10 of 11 studies, with only 2 deaths in over 3,500 patients in one large study (Russi 2024 [23]). The absolute rates are extremely low and show no meaningful variability, indicating consistent results across cohorts.

f. HFNC: Although one large study (Rogerson 2023 [29]) reported a higher mortality rate (3.6%) compared to near-zero in others, the overall direction of effect remains consistent, with all studies reporting low absolute mortality. The variability is likely explained by differences in cohort size and patient characteristics.

g. IMV: The total number of patients on IMV exceeded 6,000 across 15 studies, with over 300 deaths reported. Mortality rates were consistent in large studies and precise enough to draw stable conclusions. No serious imprecision was identified.

h. NIV: Only 2 deaths occurred among 5,042 patients receiving NIV, resulting in wide uncertainty around the mortality rate. The low number of events limits precision despite the large sample size. Even though the point estimate is reassuringly low, we cannot rule out that the true mortality might be 0.1%+.

i. HFNC: Although the total sample size is large, mortality was rare and concentrated in one study, while other studies had very small samples and no events, resulting in limited precision and wide uncertainty around effect estimates. While the total sample size is large (8,704), most deaths (63) come from one study (Rogerson [4]). Several studies had very small samples (e.g., n=13, 20, 56) and no events, meaning their mortality estimates are imprecise. The rarity of mortality events makes estimates very sensitive to small differences.

j. BIPAP: Half of the included studies (5/10) were rated as low risk of bias, while the remaining five had moderate risk primarily due to retrospective design and lack of adjustment for confounders. Ventilation failure was objectively and consistently defined across all studies, minimizing the impact of these limitations. Given the balance of risk ratings and the objective nature of the outcome, we did not downgrade for risk of bias

k. HFNC: 5/9 studies had Low Risk of Bias with strong methodology (propensity matching, multivariate adjustment, complete follow-up, secure outcome assessment). The remaining 4/9 studies, while rated Moderate, had no major fatal flaws — they still assessed mortality robustly (e.g., registry or chart review), and limitations were mostly due to design (retrospective, lack of full adjustment). None of the studies were at High risk of bias.

l. Failure rates for both HFNC and BiPAP ranged widely across studies, from 0% to over 60%, reflecting considerable numerical variation. For HFNC, studies reported failure rates from 0% (Delgado 2023 [6], Russi 2022 [7]) to 62.7% (Dunbar 2021 [35]), while BiPAP studies ranged more narrowly from 0% to 10%. Even among high-quality or large studies (e.g., Russi 2024), variability was present. However, this heterogeneity appears to reflect differences in patient severity, institutional practices, escalation thresholds, and definitions of failure, rather than conflicting treatment effects. Importantly, the direction of findings was consistent across both modalities, all studies assessed noninvasive support in pediatric asthma, and none showed contradictory or harmful results. Therefore, despite the statistical spread, the overall inconsistency was judged as not serious due to the clinical coherence of findings and explainable sources of heterogeneity.

m. The included studies evaluated BiPAP failure rates in pediatric patients with acute severe asthma, using relevant settings (PICU), populations, and outcomes. Definitions of failure were comparable across studies, and all outcomes were directly applicable to the review question. Therefore, no downgrading for indirectness

n. BiPAP: While several smaller studies had few events and likely wide confidence intervals, the pooled sample size exceeded 3400 patients. The largest study (Russi 2024 [23]) alone included over 3100 patients with a narrow failure estimate (2.4%). Overall, estimates are sufficiently precise to support a confident conclusion

o. HFNC: Most studies had adequate sample sizes, especially large cohorts like Russi 2024 [23] and Rogerson 2023 [29]. Failure events were observed in nearly all studies, reducing risk of random error. While smaller studies exist, they are balanced by larger datasets. Despite variability, the outcome (ventilation failure) was consistently captured. Overall, imprecision does not seriously lower confidence in the estimate for ventilation failure.

p. IMV: While there's no control group or statistical adjustment, both studies systematically captured adverse events using structured chart review. Follow-up was complete, and outcomes were clearly defined. Because this outcome is descriptive (not comparative) and events were passively observed rather than caused by intervention assignments, confounding is less critical

q. HFNC: Both studies had low risk of bias with adequate outcome ascertainment and follow-up.

r. NIV: While 3 of the 4 studies had moderate NOS scores, all demonstrated clear outcome ascertainment, complete follow-up, and clinically relevant definitions for adverse events. The moderate ratings were mainly due to lack of comparator groups or confounder adjustment, but these limitations are less critical for descriptive safety outcomes. Therefore, under a generous interpretation, no downgrade is applied.

s. IMV: Large difference in adverse event rates (6% vs. 70.5%) reflects differing definitions and scope of adverse events assessed (mechanical complications vs. all post-intubation events); therefore, variability is explained and does not lower confidence.

t. Both studies reported low rates of pneumothorax with HFNC; no conflicting findings.

u. NIV: While there is some variability in reported rates of adverse events, the direction of findings is consistent (all report low but present risk), and the differences are explainable by clinical and methodological diversity. Therefore, no downgrade for inconsistency.

v. Although one modality (IMV) showed serious imprecision due to small sample sizes and few events, the majority of studies (NIV and HFNC) had large cohorts with complete follow-up and consistent adverse event monitoring, supporting an overall judgment of imprecision as not serious.

w. All included studies had NOS scores ≥7/9 and demonstrated clear outcome reporting, full follow-up, and structured comparison of HFNC vs NIV

x. All studies reported effects in the same direction; no heterogeneity observed ($I^2 = 0\%$)

y. CI is narrow and does not cross the null; effect is consistent across studies and likely to influence clinical decisions.

z. Cohorts were well-defined with complete follow-up and objective outcome measurement. While no studies adjusted for confounders, all had NOS ≥5 and consistent design quality.

aa. All studies showed effects in the same direction, favoring longer LOS with intubation. Heterogeneity was low ($I^2 = 0\%$) and no conflicting findings were observed.

ab. The confidence interval is wide and includes both large benefit and harm. The result is not statistically significant, and the precision is insufficient to draw a reliable conclusion.

ac. Three of four studies had low risk of bias with well-defined cohorts, clear outcome measurement, and partial or full control for confounding. One study had moderate bias due to use of administrative data, but its weight in the meta-analysis is not dominant.

ad. Although statistical heterogeneity was high ($I^2 = 93.81\%$), the direction of effect was consistent across most studies, all favoring HFNC over standard oxygen for reduced PICU LOS. The variability observed was largely due to differences in magnitude, not direction. Furthermore, the outcome is continuous and log-transformed, which can exaggerate I^2 values.

ae. The confidence interval does not cross the line of no effect and remains within a plausible and clinically meaningful range, suggesting precise estimation of benefit.

Table S3. Certainty of Evidence Ratings for Observational Studies (GRADE Framework)

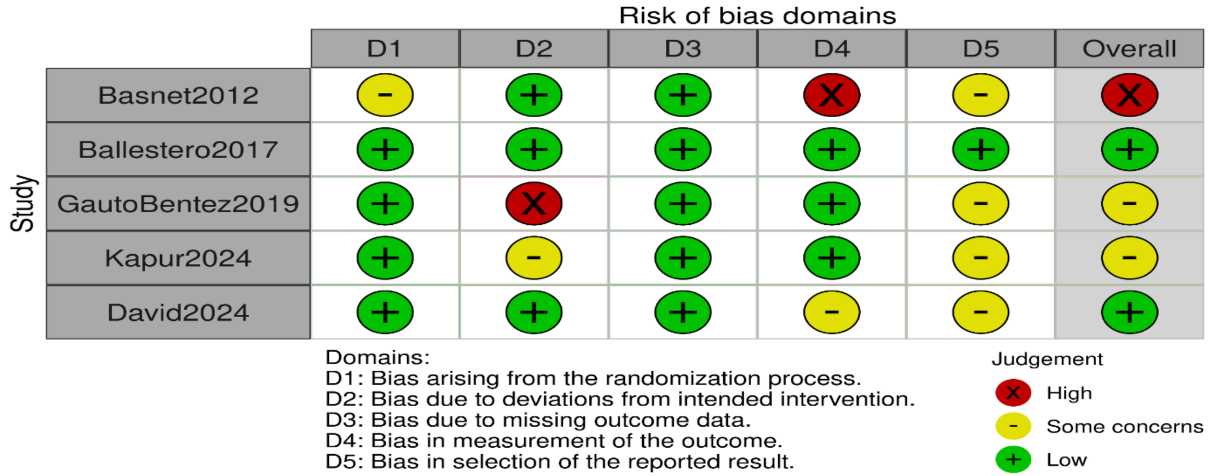

Figure S1a. Risk of bias assessment of individual randomized controlled trials using the Cochrane RoB 2.0 tool. Each study was assessed across five domains: (D1) randomization process, (D2) deviations from intended interventions, (D3) missing outcome data, (D4) outcome measurement, and (D5) selection of reported results. Judgments are categorized as low risk (green plus), some concerns (yellow dash), or high risk (red cross), with an overall risk of bias rating in the final column [14,19,20,26,27].

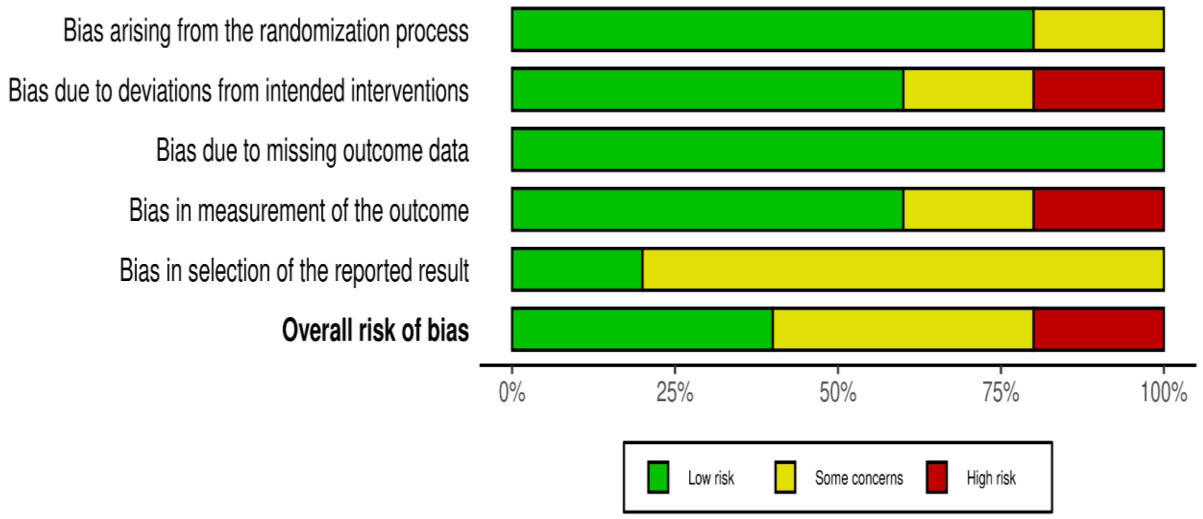

Figure S1b. Risk of Bias Summary Plot

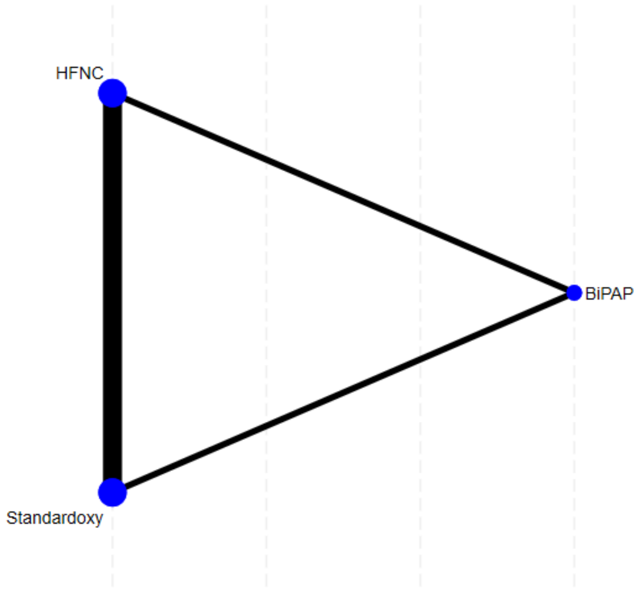

Figure S2a. Network meta-analysis map of randomized control trials between 3 modalities: standard oxygen (Standardoxy), high-flow nasal cannula (HFNC), and bilevel positive airway pressure (BiPAP).

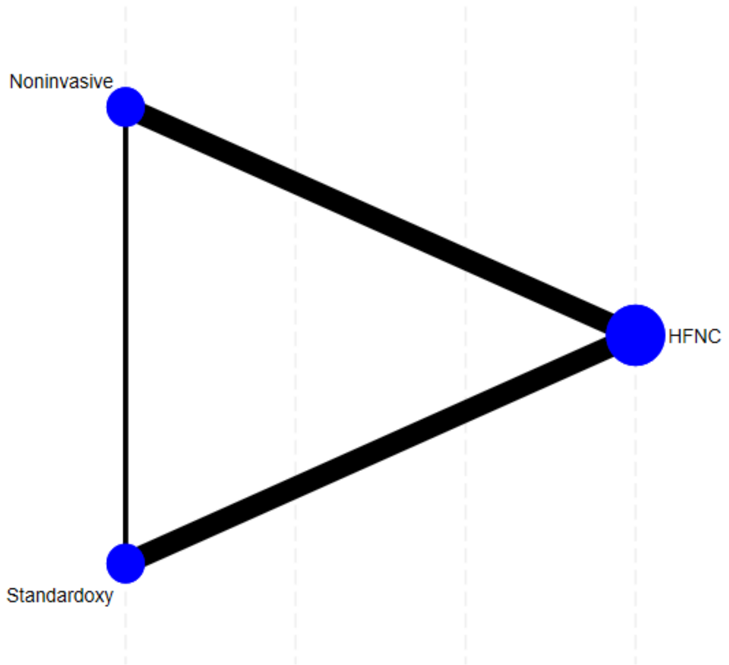

Figure S2b. Network meta-analysis map of observational studies between 3 modalities: standard oxygen (standardoxy), high-flow nasal cannula (HFNC), and non-invasive ventilation (NIV).

Year	IMV	NIV	HFNC
**2009**	18.2	1.16	0
**2010**	15	8	1
**2011**	16.45	0.9	5
**2012**	11	1.4	13
**2013**	10	3.3	16
**2014**	9.5	12	19.25
**2015**	4.5	12.5	21.9
**2016**	4.5	1	13.9
**2017**	13	14.5	32.25
**2018**	4.5	16	52.2
**2019**	8	9.15	47

Table S4. Trends in Ventilation Modalities Among PICU Patients, 2009-2019

Year	IMV	NIV	HFNC
**2009**	1.2	3.3	2.15
**2010**	3.38	4.795	6.29
**2011**	2.975	4.93	12.5
**2012**	2.685	6.98	12.37
**2013**	2.47	7.27	16.23
**2014**	0.6	2.82	16.33
**2015**	0.66	2.99	18.6
**2016**	0.59	2.6	19.655
**2017**	0.46	1.86	21.76
**2018**	0.6	2.27	29.07
**2019**	1.82	11.32	34.29

Table S5. Trends in Ventilation Modalities Among Hospitalized Patients, 2009-2019

Subgroup Pairwise Meta-Analyses

a) HFNC versus standard oxygen

Four studies were included. [1-4] HFNC was associated with significantly longer PICU stay with pooled log MD of 0.41 (95% CI 0.03 to 0.79; $p = 0.04$), corresponding to a GMR of 1.5 (95% CI: 1.03 to 2.21), as shown in Figure 3a. There was a high heterogeneity ($I^2 = 93.8\%$) and slight funnel plot asymmetry; however, Egger's test was not significant ($p = 0.068$).

b) NIV versus HFNC

Four studies were included. [2,5-7] HFNC was associated with significantly shorter PICU stay with a log MD of – 0.13 (95% CI –0.15 to –0.10; $p < 0.001$) in both fixed and random effects, and a GMR of 0.88 (95% CI: 0.86 to 0.89) as shown in Figure 5b. There was no heterogeneity ($I^2 = 0.0\%$), as evidenced by a symmetrical funnel plot and Egger's test with no publication bias ($p = 0.75$).

In summary, regarding PICU LOS, observational data suggested that NIV was associated with the longest stay and standard oxygen with the shortest stay.

c) Intubated versus non-intubated

Five studies were included. [8-12] PICU LOS was longer for intubated patients, but this was statistically non-significant with log MD of 0.52 (95% CI –0.26 to 1.30; $p = 0.19$), for both fixed and random effects, corresponding to a GMR of 1.68 (95% CI 0.93 to 3.02) as shown in Figure 5c. Low heterogeneity was observed, with no small study

effects detected. The funnel plot was symmetrical, and Egger's test showed no evidence of publication bias ($p = 0.5$). Importantly, it was often unclear what treatment was provided to the non-intubated comparison group in these studies.

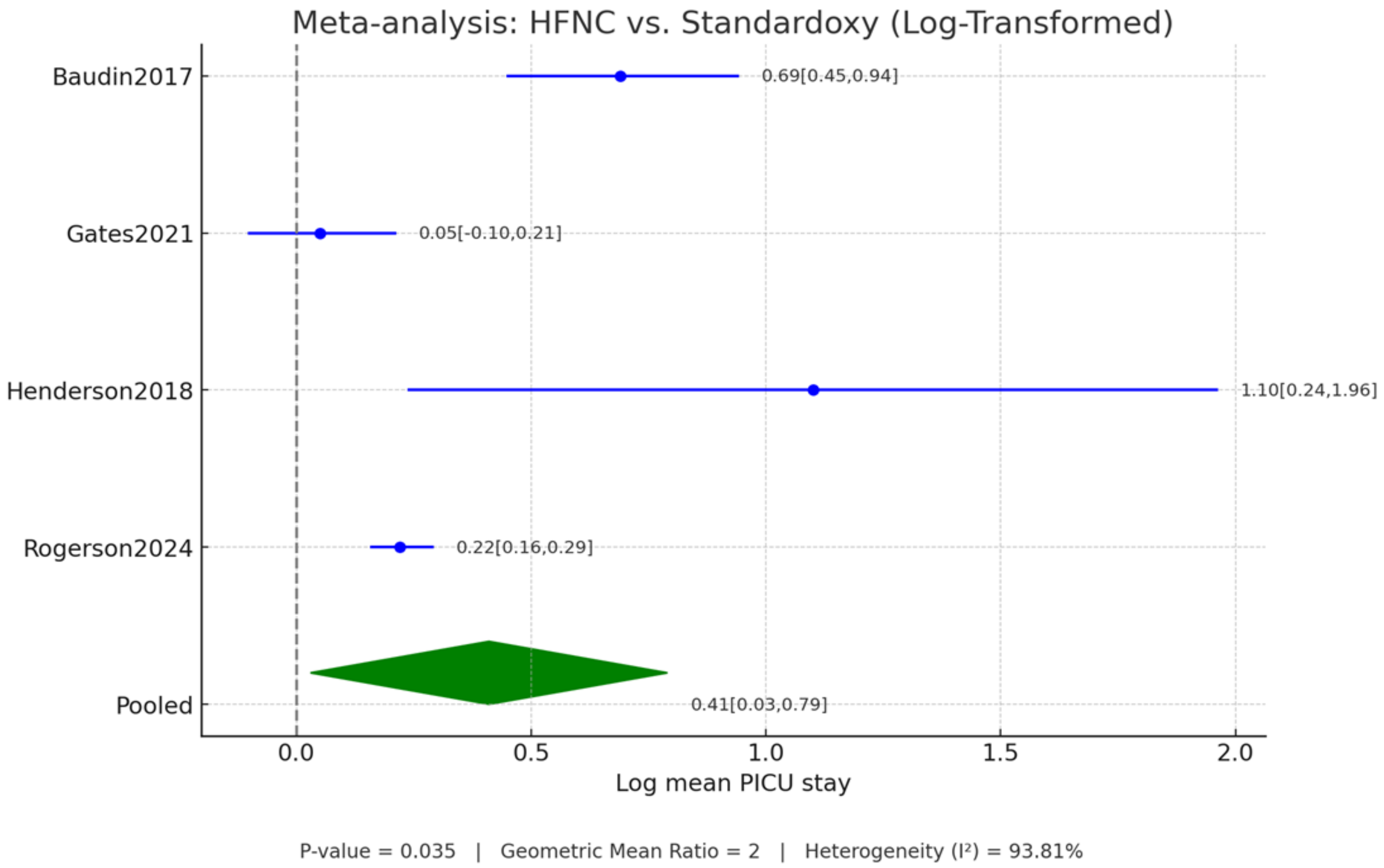

Figure S3a. Meta-analysis of PICU Length of Stay (LOS) in HFNC vs. Standard Oxygen Pediatric Asthma Patients [1,2,3,4].

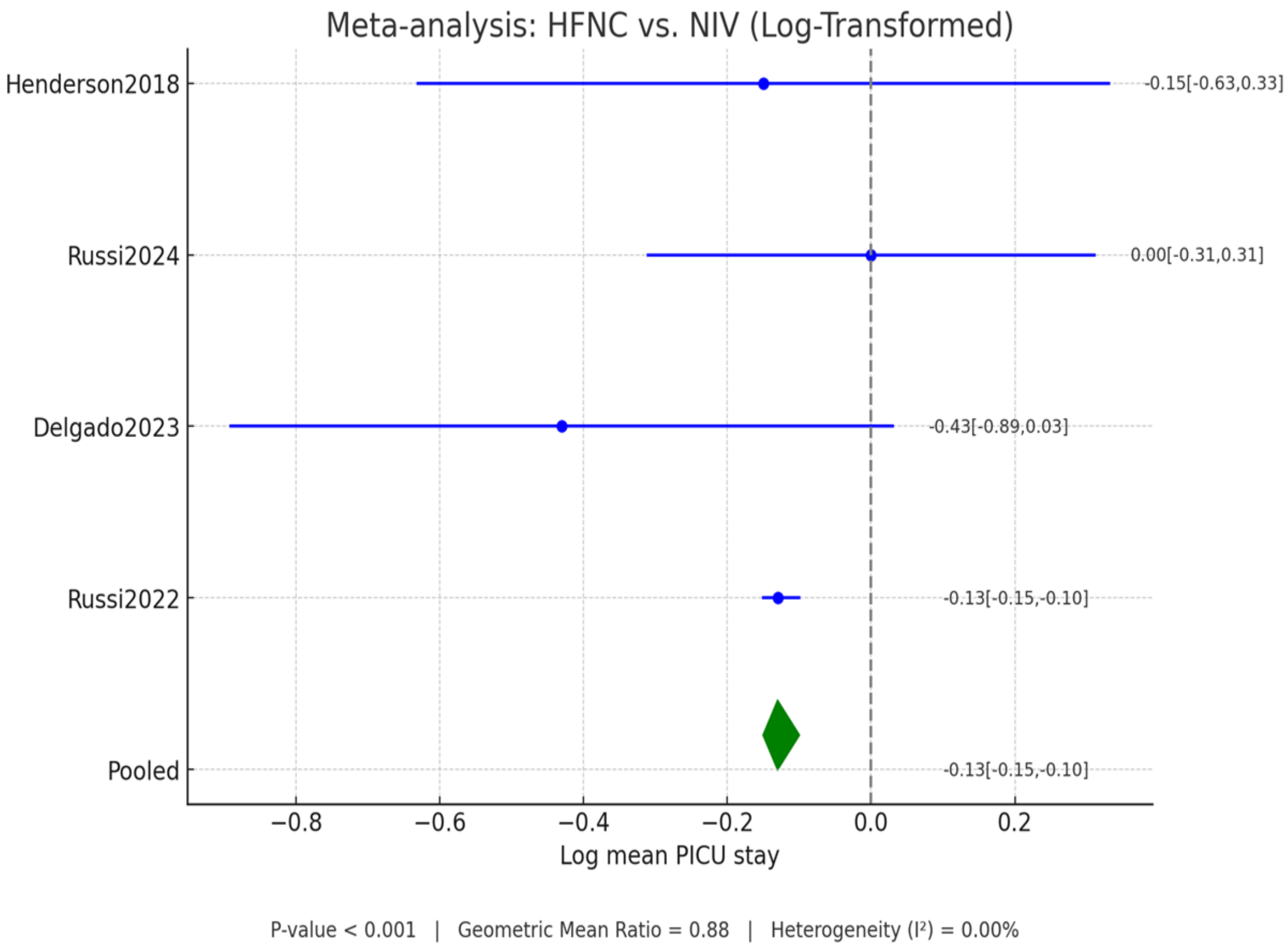

Figure S3b. Meta-analysis of PICU Length of Stay (LOS) in HFNC vs. NIV Pediatric Asthma Patients [2,6,7,23].

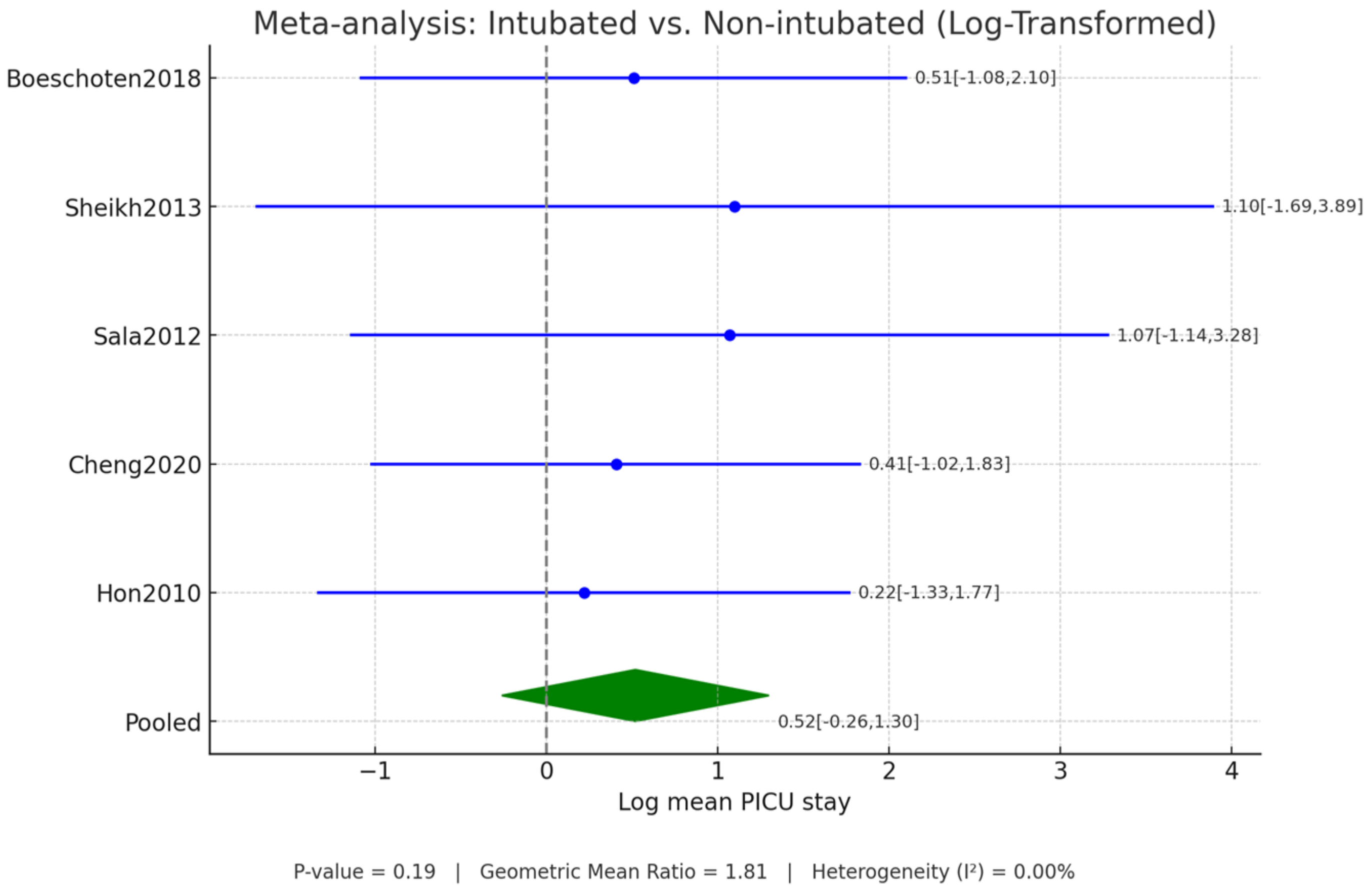

Figure S3c. Meta-analysis of PICU Length of Stay (LOS) in Intubated vs. Non-intubated Pediatric Asthma Patients [8,9,10,11,12].

Rank/Treatment	NIV(BiPAP)	HFNC	Standard oxygen
Best	90.8%	6.3%	2.6%
2nd	6.5%	63.4%	30.0%
Worst	2.7%	30.3%	67.0%

Table S6a RCT Network Meta-analysis of RCTs: Ranking Each Treatment for asthma score improvement **(**Estimated probabilities (%) for each treatment rank, assuming lower asthma scores indicate better outcomes)

Rank/ Treatment	NIV	HFNC	Standard oxygen
Best	1.4%	18.8%	79.8%
2nd	10%	75.2%	14.8%
Worst	88.6%	6%	5.4%

Table S6b Network Meta-analysis for observational studies: Ranking of treatments based of PICU length of stay (Estimated probabilities (%) of each treatment rank, assuming shorter PICU stay indicated better outcome)

Funnel Plots:

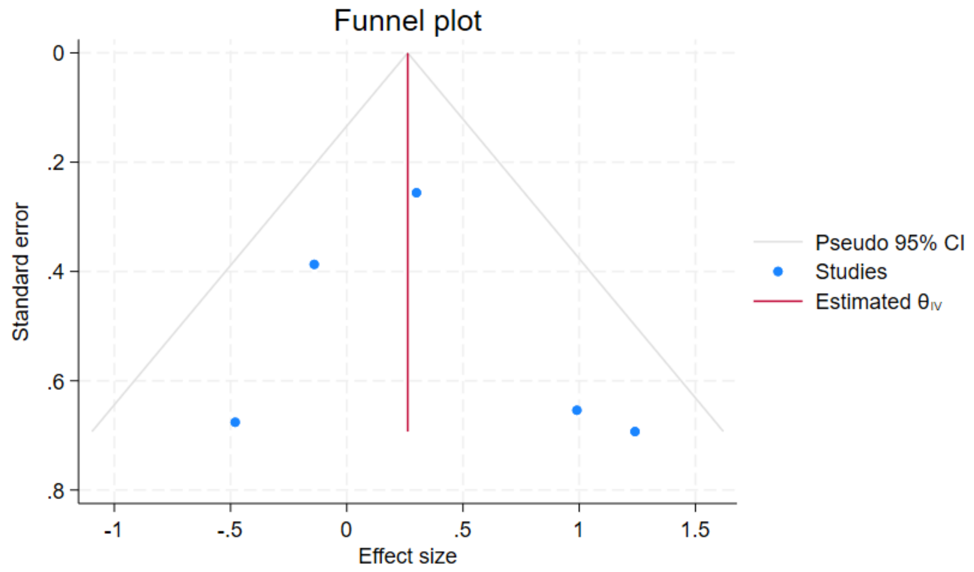

Figure S4a. Funnel plot of network randomized control trials. Egger's bias p = 0.53.

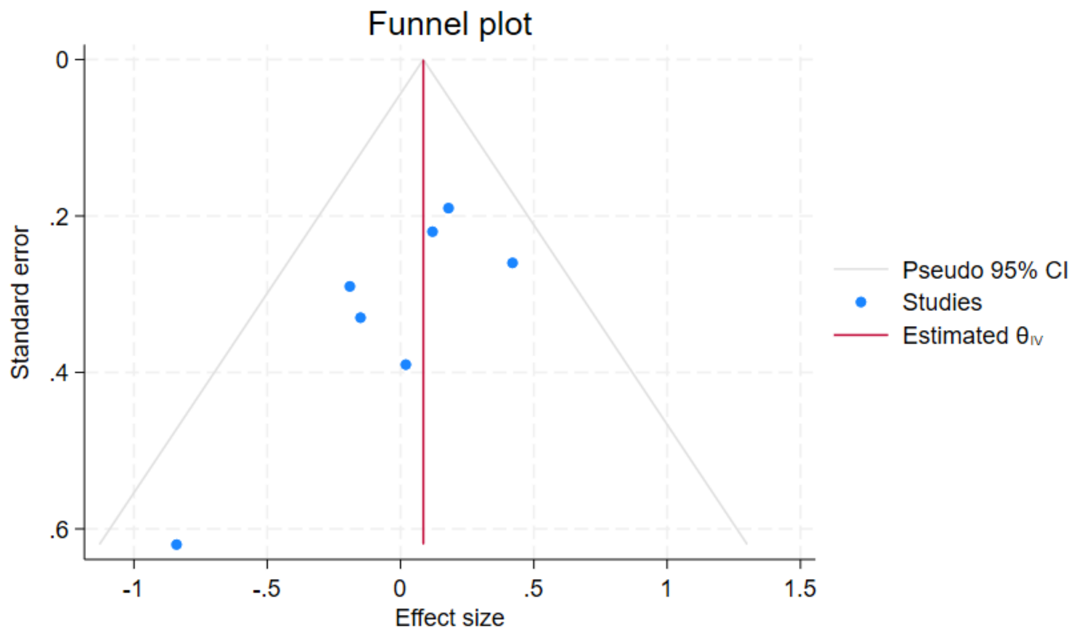

Figure S4b. Funnel plot of network observational studies. Egger's bias p = 0.09.

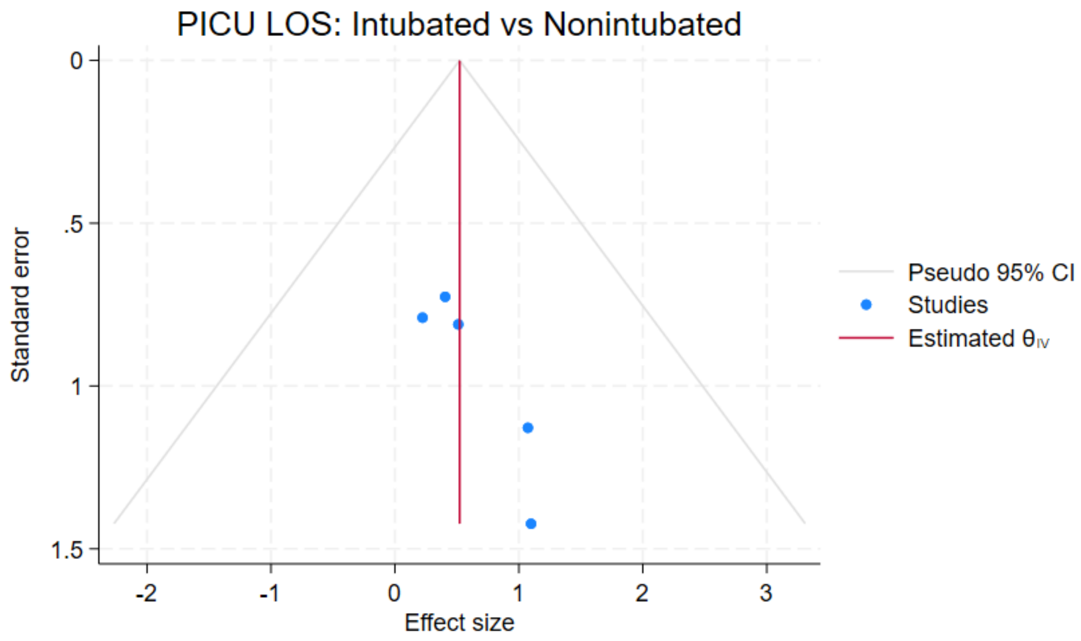

Figure S4c. Funnel plot, intubated versus non-intubated. Egger's bias p = 0.5

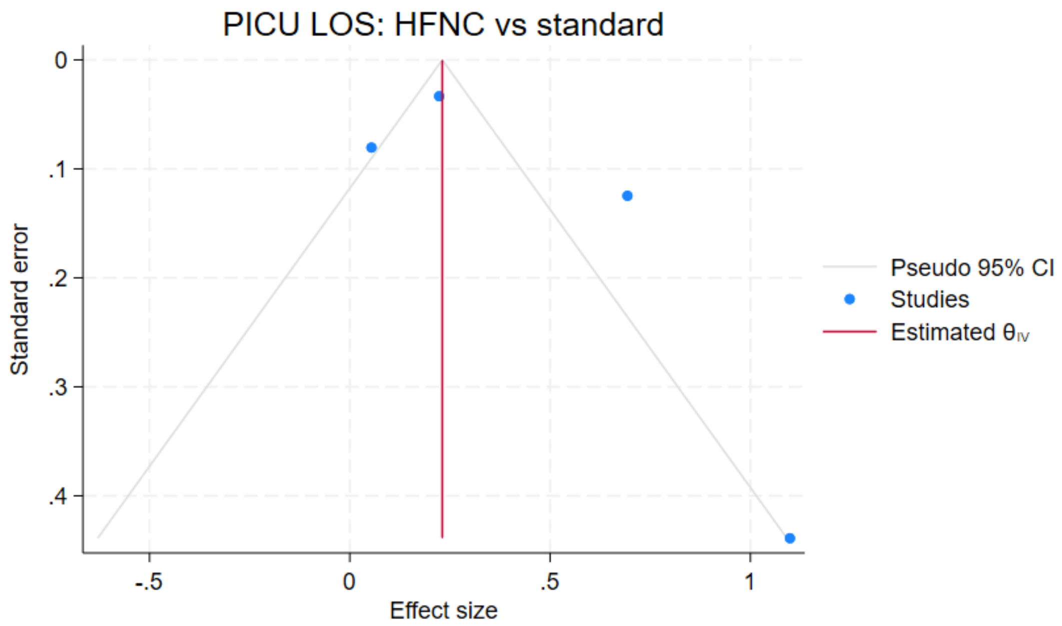

Figure S4d. Funnel plot, HFNC versus standard oxygen, Egger's bias p = 0.068

HFNC vs. NIV

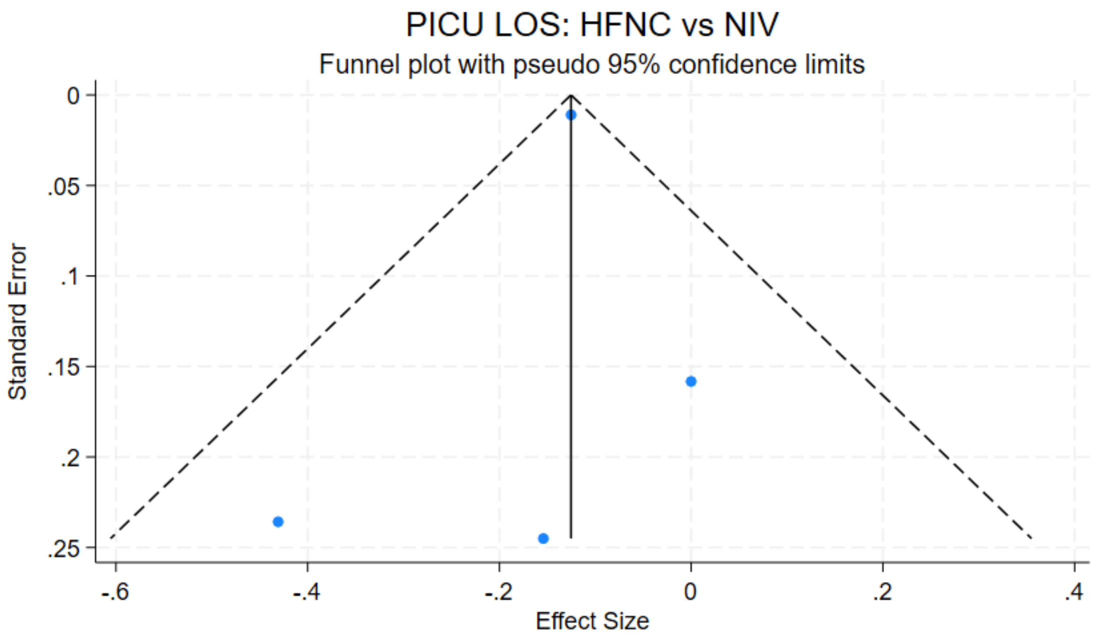

Figure S4e. Funnel plot, HFNC versus NIV. Egger's bias p = 0.75

Study Name	BiPAP Patients	BiPAP Failure
**Russi 2024 [23]**	3164	76
**Pilar 2017 [30]**	22	0
**Russi 2022 [7]**	26	0
**Delgado 2023 [6]**	6	0
**Usala 2022 [31]**	24	1
**Abramo 2017 [15]**	62	6
**Al-Eydhy 2015 [32]**	24	0
**Basnet 2012 [14]**	10	1
**William 2011 [25]**	10	1
**Mayordomo 2011 *episodes [16]**	72	5
**Kang 2020 *Episodes [17]**	25	1

Table S7. Use and Failure Rates of BiPAP in Included Studies.

Study Name	HFNC Patients	HFNC failure
**GATES 2021 [3]**	104	11
**Baudin 2017 [1]**	39	2
**Russi 2024 [23]**	6,562	483
**Pilar 2017 [30]**	20	8
**Russi 2022 [7]**	13	0
**Delgado 2023 [6]**	56	0
**David 2024 [20]**	25	1
**Dunbar 2021 [35]**	51	32
**Kapur 2024 [27]**	28	18
**Rogerson 2024 [4]**	443	60
**Colin Rogerson 2023 [29]**	1766	534

Table S8. High-Flow Nasal Cannula (HFNC) Use and Failure Rates Across HFNC Studies.

Name	IMV (n)	Death (n)
**Hartman 2010 [22]**	439	12
**Smith MA. 2023 [5]**	3,123	235
**Craig 2022 [34]**	4	0
**Salie 2025 [43]**	18	0
**Monteverde-Fernandez 2021 [44]**	27	0
**Rampa 2015 [37]**	1,376	55
**Rampersad 2018 [24]**	41	3
**Shibata 2014 [36]**	157	3
**Newth 2012 [21]**	260	10
**Mukherjee 2022 [42]**	828	14
**Deho 2010 [33]**	51	0
**Cheng 2020 [12]**	7	0
**Hon 2010 [9]**	6	0
**Sheikh 2013 [10]**	17	0
**Abramo 2017 [15]**	6	0

Table S9. Mortality in Pediatric Asthma Patients Receiving IMV

Name	NIV (n)	Death (n)
**Russi 2022 [7]**	26	0
**Al-Eyadhy 2015 [32]**	24	0
**Pilar 2017 [30]**	22	0
**Kang 2020 [17]**	25	0
**Delgado 2023 [6]**	6	0
**Craig 2022 [34]**	20	0
**Abramo 2017 [15]**	1157	0
**Usala 2022 [31]**	54	0
**Salie 2025 [43]**	33	0
**Russi 2024 [23]**	3,521	2
**Monteverde-Fernandez 2021 [44]**	154	0

Table S10. Mortality in Pediatric Asthma Patients Receiving NIV

Name	HFNC (n)	death (n)
**Russi 2022 [7]**	13	0
**Pilar 2017 [30]**	20	0
**Delgado 2023 [6]**	56	0
**Rogerson 2023 [29]**	1,766	63
**Salie 2025 [43]**	92	0
**Russi 2024 [23]**	6,562	4
**Monteverde-Fernandez 2021 [44]**	185	0

Table S11. Mortality in Pediatric Asthma Patients Receiving HFNC.

Heart Rate and Respiratory Rate Improvement Across Ventilation Strategies

a) Non-invasive ventilation

CPAP was associated with significant heart rate (HR) and respiratory rate (RR) reduction in one study. [13] BiPAP significantly reduced RR in three studies [14-16],and HR in two. [14,16] Another study reported a post-treatment HR improvement favoring BiPAP, but without statistical testing. [17]

b) High-flow nasal cannula

Three studies found a significant decrease in HR and RR [1,4,18], whereas two studies found no significant changes. [14,19]

c) HFNC vs. BiPAP

One trial comparing HFNC and BiPAP showed that both groups experienced significant improvements in both HR and RR, with no statistically significant differences observed between groups. [20]

Adverse Events Associated with Ventilation Modalities

Two studies reported complications associated with IMV: one described barotrauma in eight patients, while the other study reported hypotension, severe bronchospasm, lung collapse, and pneumothorax. [21,22]

Five studies described adverse events with NIV. One study noted nasal bridge pain for two patients, [14] two studies reported pneumothoraxes in 14 and one patient, respectively. [23,24] One article reported one case of vomiting.[25] The other reported minor adverse events including skin irritation, gastric distension, bleeding, and subcutaneous emphysema. [10]

For HFNC, two studies reported pneumothorax: one case in one study and 25 in the other. [1,23]

1. Baudin, F.; Buisson, A.; Vanel, B.; Massenavette, B.; Pouyau, R.; Javouhey, E. Nasal high flow in management of children with status asthmaticus: A retrospective observational study. Ann. Intensive Care 2017, 7, 55
2. Henderson, M.B.; Schunk, J.E.; Henderson, J.L.; Larsen, G.Y.; Wilkes, J.; Bratton, S.L. An assessment of asthma therapy in the pediatric ICU. Hosp. Pediatr. 2018, 8, 361–367
3. Gates, R.M.; Haynes, K.E.; Rehder, K.J.; Zimmerman, K.O.; Rotta, A.T.; Miller, A.G. High-flow nasal cannula in pediatric critical asthma. Respir. Care 2021, 66, 1240–1246
4. Rogerson, C.; AbuSultaneh, S.; Sanchez-Pinto, L.N.; Gaston, B.; Wiehe, S.; Schleyer, T.; et al. A matched analysis of the use of high-flow nasal cannula for pediatric severe acute asthma. Pediatr. Pulmonol. 2024, 59, 3457–3466
5. Smith, M.A.; Dinh, D.; Ly, N.P.; Ward, S.L.; McGarry, M.E.; Zinter, M.S. Changes in the use of invasive and non-invasive mechanical ventilation in pediatric asthma: 2009–2019. Ann. Am. Thorac. Soc. 2023, 20, 245–253
6. Delgado, C.S.; García-Salido, A.; García-Teresa, M.A.; Azagra-Garde, A.M.; Leoz-Gordillo, I.; Caro-Patón, G.L.; Nieto-Moro, M. High-flow nasal cannula and non-invasive mechanical ventilation in pediatric asthma exacerbation: Two-year prospective observational study in intensive care. Bol. Med. Hosp. Infant. Mex. 2023, 80, 355–360
7. Russi, B.W.; Lew, A.; McKinley, S.D.; Morrison, J.M.; Sochet, A.A. High-flow nasal cannula and bilevel positive airway pressure for pediatric status asthmaticus: A single-center, retrospective descriptive and comparative cohort study. J. Asthma 2022, 59, 757–764
8. Sala, K.A.; Karamessinis, L.; Zucker, A.R.; Carroll, C.L. Respiratory failure in children with status asthmaticus: A review of the VPS database. Pediatr. Allergy Immunol. Pulmonol. 2012, 25, 226–230
9. Hon, K.L.; Tang, W.S.; Leung, T.F.; Cheung, K.L.; Ng, P.C. Outcome of children with life-threatening asthma necessitating pediatric intensive care. Ital. J. Pediatr. 2010, 36, 47
10. Sheikh, S.; Khan, N.; Ryan-Wenger, N.A.; McCoy, K.S. Demographics, clinical course, and outcomes of children with status asthmaticus treated in a pediatric intensive care unit: An 8-year review. J. Asthma 2013, 50, 364–369.
11. Boeschoten, S.A.; Buysse, C.M.P.; Merkus, P.J.F.M.; van Wijngaarden, J.M.C.; Heisterkamp, S.G.J.; de Jongste, J.C.; et al. Children with severe acute asthma admitted to Dutch PICUs: A changing landscape. Pediatr. Pulmonol. 2018, 53, 857–865
12. Cheng, W.T.; Hon, K.L.; Chan, R.W.Y.; Chan, L.C.N.; Wong, W.; Cheung, H.M.; Qian, S.Y. Outcome of status asthmaticus at a pediatric intensive care unit in Hong Kong. Clin. Respir. J. 2020, 14, 462–470
13. de Freitas Dantas Gomes, E.L.; Costa, D.; Germano, S.M.; Borges, P.V.; Sampaio, L.M. Effects of CPAP on clinical variables and autonomic modulation in children during an asthma attack. Respir. Physiol. Neurobiol. 2013, 188, 66–70
14. Basnet, S.; Mander, G.; Andoh, J.; Klaska, H.; Verhulst, S.; Koirala, J. Safety, efficacy, and tolerability of early initiation of noninvasive positive pressure ventilation in pediatric patients admitted with status asthmaticus: A pilot study. Pediatr. Crit. Care Med. 2012, 13, 393–398.

15. Abramo, T.; Williams, A.; Mushtaq, S.; Meredith, M.; Sepaule, R.; Crossman, K.; et al. Paediatric ED BiPAP continuous quality improvement programme with patient analysis: 2005–2013. BMJ Open 2017, 7, e011845
16. Mayordomo-Colunga, J.; Medina, A.; Rey, C.; Concha, A.; Menéndez, S.; Arcos, M.L.; et al. Non-invasive ventilation in pediatric status asthmaticus: A prospective observational study. Pediatr. Pulmonol. 2011, 46, 949–955
17. Kang, C.M.; Wu, E.T.; Wang, C.C.; Lu, F.; Chiang, B.L.; Yen, T.A. Bilevel positive airway pressure ventilation efficiently improves respiratory distress in initial hours treating children with severe asthma exacerbation. J. Formos. Med. Assoc. 2020, 119, 1415–1421
18. González Martínez, F.; González Sánchez, M.I.; Toledo Del Castillo, B.; Pérez Moreno, J.; Medina Muñoz, M.; Rodríguez Jiménez, C.; Rodríguez Fernández, R. Treatment with high-flow oxygen therapy in asthma exacerbations in a paediatric hospital ward: Experience from 2012 to 2016. An. Pediatr. (Engl. Ed.) 2019, 90, 72–78
19. Ballestero, Y.; De Pedro, J.; Portillo, N.; Martinez-Mugica, O.; Arana-Arri, E.; Benito, J. Pilot clinical trial of high-flow oxygen therapy in children with asthma in the emergency service. J. Pediatr. 2018, 194, 204–210.e3
20. David, M.M.C.; Gomes, E.L.F.D.; Cavassini, C.L.F.; Luiz, J.G.; Costa, D. Comparison of the effects of high-flow nasal cannula and bilevel positive airway pressure treatments as respiratory physiotherapy interventions for children with asthma exacerbation: A randomized clinical trial. Einstein (Sao Paulo) 2024, 22, eAO0588
21. Newth, C.J.; Meert, K.L.; Clark, A.E.; Moler, F.W.; Zuppa, A.F.; Berg, R.A.; et al. Fatal and near-fatal asthma in children: The critical care perspective. J. Pediatr. 2012, 161, 214–221.e3
22. Hartman, M.E.; Linde-Zwirble, W.T.; Angus, D.C.; Watson, R.S. Trends in admissions for pediatric status asthmaticus in New Jersey over a 15-year period. Pediatrics 2010, 126, e904–e911
23. Russi, B.W.; Roberts, A.R.; Nievas, I.F.; Rogerson, C.M.; Morrison, J.M.; Sochet, A.A. Noninvasive respiratory support for pediatric critical asthma: A multicenter cohort study. Respir. Care 2024, 69, 534–540.
24. Rampersad, N.; Wilkins, B.; Egan, J.R. Outcomes of paediatric critical care asthma patients. J. Paediatr. Child Health 2018, 54, 633–637
25. Williams, A.M.; Abramo, T.J.; Shah, M.V.; Miller, R.A.; Burney Jones, C.; et al. Safety and clinical findings of BiPAP utilization in children 20 kg or less for asthma exacerbations. Intensive Care Med. 2011, 37, 1338–1343
26. Gauto Benítez, R.; Morilla Sanabria, L.P.; Pavlicich, V.; Mesquita, M. High-flow nasal cannula oxygen therapy in patients with asthmatic crisis in the pediatric emergency department. *Rev. Chil. Pediatr.* **2019**, *90*, 642–648.
27. Kapur, A.; Rojas-Anaya, H.; Roberts, G.; Roland, D.; Gupta, A.; Lazner, M.; Bayreuther, J.; Cantle, F.; Jones, C.; Pappachan, J.; et al. High-flow humidified oxygen as an early intervention in children with acute severe asthma: A feasibility randomised controlled trial. *ERJ Open Res.* **2024**, *10*, 00168–02024.
28. González Martínez, F.; González Sánchez, M.I.; Toledo Del Castillo, B.; Pérez Moreno, J.; Medina Muñoz, M.; Rodríguez Jiménez, C.; Rodríguez Fernández, R. Treatment with high-flow oxygen therapy in asthma exacerbations in a paediatric hospital ward: Experience from 2012 to 2016. *An. Pediatr. Engl. Ed.* **2019**, *90*, 72–78.
29. Rogerson, C.; Owora, A.; He, T.; Carroll, A.; Schleyer, T.; AbuSultaneh, S.; Tu, W.; Mendonca, E. High-flow nasal cannula use is associated with increased hospital length of stay for pediatric asthma. *Pediatr. Pulmonol.* **2023**, *58*, 3046–3053.

30. Pilar, J.; Modesto, I.; Alapont, V.; Lopez-Fernandez, Y.M.; Lopez-Macias, O.; Garcia-Urabayen, D.; Amores-Hernandez, I. High-flow nasal cannula therapy versus non-invasive ventilation in children with severe acute asthma exacerbation: An observational cohort study. *Med. Intensiva* **2017**, *41*, 418–424.
31. Usala, C.; Wilson, P. Noninvasive ventilation use in pediatric status asthmaticus. *J. Asthma* **2022**, *59*, 1338–1342.
32. Al-Eyadhy, A.A.; Temsah, M.H.; Alhaboob, A.A.; Aldubayan, A.K.; Almousa, N.A.; Alsharidah, A.M.; Alangari, M. Asthma changes at a pediatric intensive care unit after 10 years: Observational study. *Ann. Thorac. Med.* **2015**, *10*, 243–248.
33. Dehò, A.; Lutman, D.; Montgomery, M.; Petros, A.; Ramnarayan, P. Emergency management of children with acute severe asthma requiring transfer to intensive care. *Emerg. Med. J.* **2010**, *27*, 834–837.
34. Craig, S.; Powell, C.V.E.; Nixon, G.M.; Oakley, E.; Hort, J.; Armstrong, D.S.; Ranganathan, S.; Kochar, A.; Wilson, C.; George, S.; et al. Treatment patterns and frequency of key outcomes in acute severe asthma in children: A Paediatric Research in Emergency Departments International Collaborative (PREDICT) multicentre cohort study. *BMJ Open Respir. Res.* **2022**, *9*, e001137.
35. Dunbar, K.S.; Fox, S.N.; Thomas, J.F.; Brittan, M.S.; Soskolne, G.; Cotter, J.M. When to transfer: Predictors of pediatric high-flow nasal cannula failure at a community hospital. *Hosp. Pediatr.* **2024**, *14*, 45–51.
36. Shibata, S.; Khemani, R.G.; Markovitz, B.P. Patient origin is associated with duration of endotracheal intubation and PICU length of stay for children with status asthmaticus. *J. Intensive Care Med.* **2014**, *29*, 154–159.
37. Rampa, S.; Allareddy, V.; Asad, R.; Nalliah, R.P.; Allareddy, V.; Rotta, A.T. Outcomes of invasive mechanical ventilation in children and adolescents hospitalized due to status asthmaticus in the United States: A population-based study. *J. Asthma* **2015**, *52*, 423–430.
38. de Miguel-Díez, J.; Jiménez-García, R.; Hernández-Barrera, V.; López de Andrés, A.; Villa-Asensi, J.R.; Plaza, V.; Carrasco-Garrido, P. National trends in hospital admissions for asthma exacerbations among pediatric and young adult populations in Spain (2002–2010). *Respir. Med.* **2014**, *108*, 983–991.
39. Gutierrez-Albaladejo, N.; Jimenez-Garcia, R.; Albaladejo-Vicente, R.; Villanueva-Orbaiz, R.; de Miguel-Diez, J.; Noriega, C.; Lopez-De-Andres, A. Trends in hospital admissions among children with asthma in Spain (2011–2020). *Eur. J. Pediatr.* **2023**, *182*, 2409–2419.
40. Smith, A.; França, U.L.; McManus, M.L. Trends in the use of noninvasive and invasive ventilation for severe asthma. *Pediatrics* **2020**, *146*, e20200534.
41. Lee, D.S.; Gross, E.; Hotz, A.; Rastogi, D. Comparison of severity of asthma hospitalization between African American and Hispanic children in the Bronx. *J. Asthma* **2020**, *57*, 736–742.
42. Mukherjee, M.; Cunningham, S.; Bhuia, M.R.; Lo, T.M.; Been, J.V.; Sheikh, A. Asthma in paediatric intensive care in England residents: An observational study. *Sci. Rep.* **2022**, *12*, 1315.
43. Salie, M.; Salie, S. A retrospective review of children admitted with acute severe asthma to the paediatric intensive care unit, Red Cross War Memorial Children's Hospital between 2009 and 2019. *J. Paediatr. Child Health* **2025**, *61*, 441–446.
44. Monteverde-Fernandez, N.; Diaz-Rubio, F.; Vásquez-Hoyos, P.; Rotta, A.T.; González-Dambrauskas, S.; LARed Network. Variability in care for children with severe acute asthma in Latin America. *Pediatr. Pulmonol.* **2021**, *56*, 384–391.

45. Hasegawa, K.; Tsugawa, Y.; Brown, D.F.; Camargo, C.A., Jr. Childhood asthma hospitalizations in the United States, 2000–2009. *J. Pediatr.* **2013**, *163*, 1127–1133.e3.
